# *In silico* assessment of histotripsy-induced changes in catheter-directed thrombolytic delivery

**DOI:** 10.3389/fphys.2023.1225804

**Published:** 2023-06-28

**Authors:** Kenneth B. Bader, Katia Flores Basterrechea, Samuel A. Hendley

**Affiliations:** ^1^ Department of Radiology, University of Chicago, Chicago, IL, United States; ^2^ University of Nebraska Medical Center, Omaha, NE, United States

**Keywords:** histotripsy, catheter-directed thrombolytics, Monte Carlo simulation, ablation, venous thrombosis, diffusion

## Abstract

**Introduction:** For venous thrombosis patients, catheter-directed thrombolytic therapy is the standard-of-care to recanalize the occluded vessel. Limitations with thrombolytic drugs make the development of adjuvant treatments an active area of research. One potential adjuvant is histotripsy, a focused ultrasound therapy that lyses red blood cells within thrombus via the spontaneous generation of bubbles. Histotripsy has also been shown to improve the efficacy of thrombolytic drugs, though the precise mechanism of enhancement has not been elucidated. In this study, *in silico* calculations were performed to determine the contribution of histotripsy-induced changes in thrombus diffusivity to alter catheter-directed therapy.

**Methods:** An established and validated Monte Carlo calculation was used to predict the extent of histotripsy bubble activity. The distribution of thrombolytic drug was computed with a finite-difference time domain (FDTD) solution of the perfusion-diffusion equation. The FDTD calculation included changes in thrombus diffusivity based on outcomes of the Monte Carlo calculation. Fibrin degradation was determined using the known reaction rate of thrombolytic drug.

**Results:** In the absence of histotripsy, thrombolytic delivery was restricted in close proximity to the catheter. Thrombolytic perfused throughout the focal region for calculations that included the effects of histotripsy, resulting in an increased degree of fibrinolysis.

**Discussion:** These results were consistent with the outcomes of *in vitro* studies, suggesting histotripsy-induced changes in the thrombus diffusivity are a primary mechanism for enhancement of thrombolytic drugs.

## Introduction

Venous thromboembolism remains a major source of morbidity and mortality worldwide (Tsao et al., 2022). For critical obstructions, catheter-delivery of thrombolytic drugs is the frontline approach to restore flow in the afflicted vessel (Chen et al., 2016). There are a number of limitations to catheter-directed therapies, including variable outcome based on thrombus age (Czaplicki et al., 2017), and complications that scale with dose and treatment duration (Mewissen et al., 1999). Mechanical thrombectomy devices for venous thrombosis show promise, but are still are under investigation (Quinn et al., 2021; Yuriditsky et al., 2021; Abramowitz et al., 2023). A potential adjuvant to improve outcomes for catheter-directed thrombolytics is histotripsy, a focused ultrasound therapy that relies on the spontaneous generation of bubbles to ablate tissue without heating (Bader et al., 2019). Histotripsy is under development for the treatment of several aliments (Khokhlova et al., 2015; Xu et al., 2021), including thrombosis (Maxwell et al., 2009; Bader et al., 2016; Khokhlova et al., 2016; Pandey et al., 2020).

Prior studies have demonstrated histotripsy is effective at breaking down cells, but has limited efficacy for extracellular structures (Vlaisavljevich et al., 2013; Wang et al., 2013) such as fibrin found within thrombus (Hendley et al., 2022). Recent investigations have combined histotripsy with a thrombolytic therapy that degrades the fibrin network to address the entire thrombus structure (Bollen et al., 2020). These studies indicate histotripsy increases the efficacy of the thrombolytic drug (Hendley et al., 2021a), though the precise reason for enhancement is not well understood. One potential mechanism may be changes in the thrombus diffusivity due to histotripsy exposure. The activity of thrombolytic drugs is limited in close proximity to the catheter due to the combined effects of the dense thrombus structure (Wufsus et al., 2013) and fibrinolytic inhibitors (Shibeko et al., 2020). Histotripsy has been shown to increase the diffusivity of thrombus by a factor of 10 (Allen et al., 2017), which may improve delivery of thrombolytic.

The objective of this *in silico* study was to assess the role of histotripsy-induced changes in thrombus diffusivity on the delivery profile of catheter-directed thrombolytic drugs. Regions of thrombus damage due to bubble activity were determined with Monte Carlo calculations (Maxwell et al., 2013) and an analytic model of bubble expansion (Bader and Holland, 2016; Bader, 2018). Both the Monte Carlo calculation and analytic model have been validated in previous studies. Spatial variability in the bubble dynamics based on thrombus composition were accounted for by assigning each pixel in the calculation to be fibrin or red blood cell using annotated histological sections of *ex vivo* specimens (Hendley et al., 2021b). A finite difference, time domain (FDTD) calculation was performed to assess the delivery of thrombolytic drug from an infusion catheter. The FDTD calculation used spatially-varying diffusive properties to account for changes in thrombus structure due to histotripsy exposure based on outcomes of the Monte Carlo calculation (Allen et al., 2017).

## Methods

### Model overview

**FIGURE 1:**
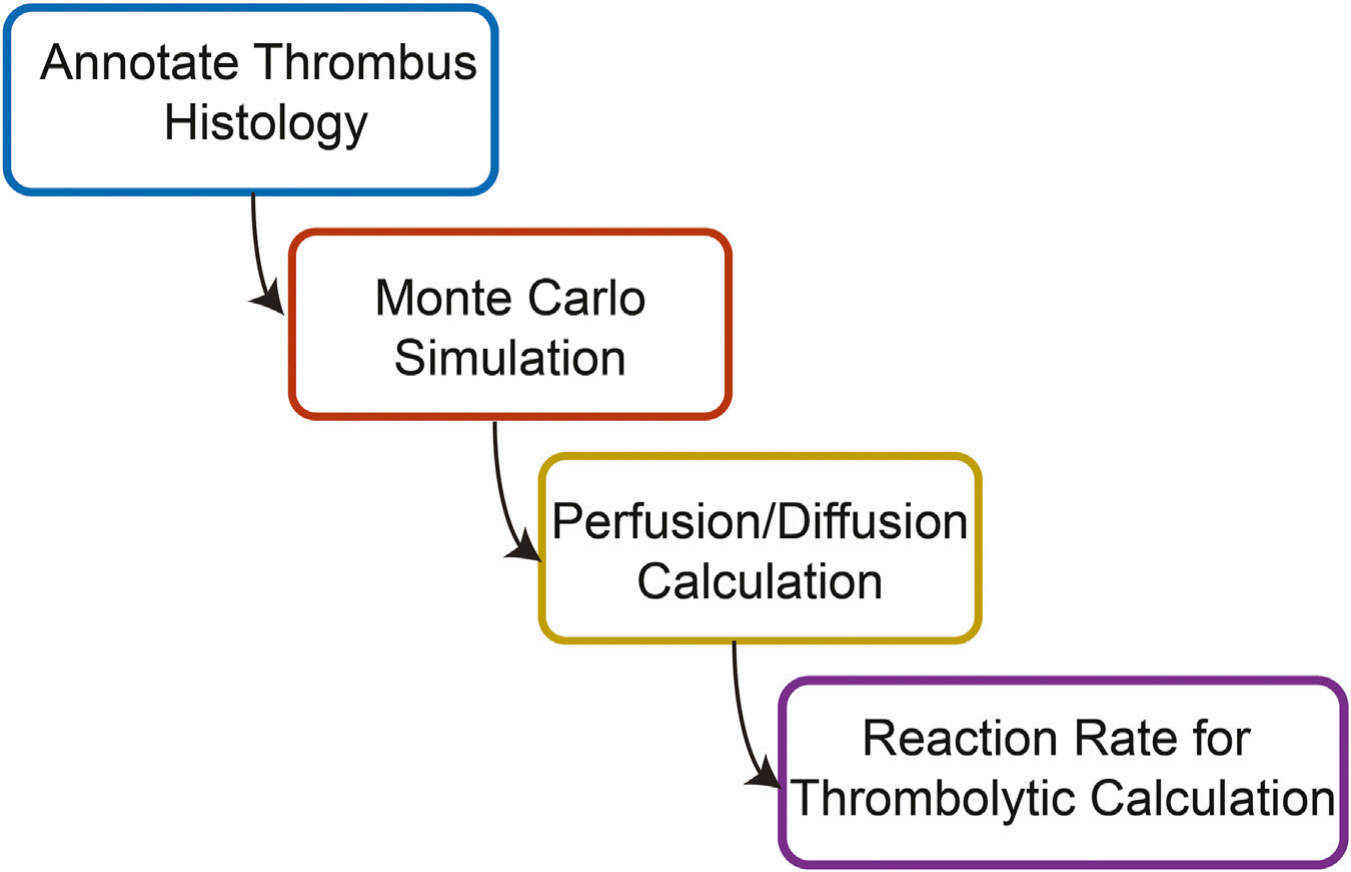
Overview of the steps used to compute the combined effects of histotripsy ablation and thrombolytic drug on venous thrombus degradation.

An overview of the steps for calculations in this study is shown in Figure 1. Each section of the calculation was performed serially. This protocol assumes each step in the calculation is an independent process. Therefore, any processes that may occur during the simultaneous administration of histotripsy and catheter-directed therapy are neglected. Further information for each of these steps of the calculation are outlined below.

### Histology image processing

**FIGURE 2:**
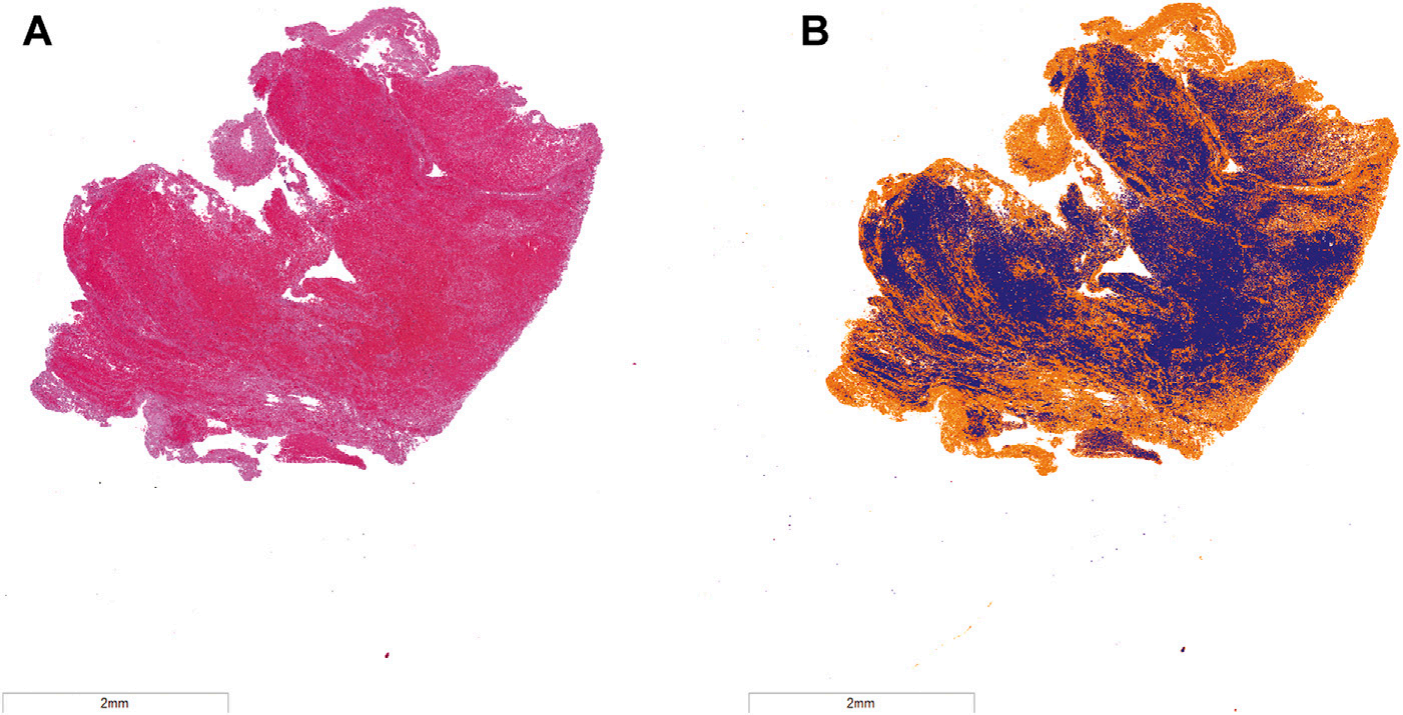
**(A)** Hematoxylin and eosin (H&E) stain of representative venous thrombus. **(B)** Color deconvolution of H&E image using the Positive Pixel Algorithm. Fibrin is noted in orange, and red blood cells are in blue. The bar in the lower left corner corresponds to a 2 mm distance.

Samples of venous thrombi were collected from twenty two volunteer patients that underwent mechanical thrombectomy at the University of Chicago Medical Center between December 2018 and December 2020 (University of Chicago IRB #18-0179) (Hendley et al., 2021b). Demographics for the volunteer patients are listed in Table 1. Following collection, samples were fixed, paraffin embedded, sectioned to 5-µm thickness, and stained with Hematoxylin and Eosin (H&E, Tissue-Tek Prisma H&E Stain Kit #1, Sakura Finetek USA, Inc., Torrance, CA, United States). Stained specimen were scanned at ×20 magnification (ScanScope XT, Leica Biosystems, Wetzlar, Germany), and analyzed with the Positive Pixel Algorithm (ScanScope XT, Leica Biosystems, Wetzlar, Germany) to identify red blood cells and fibrin (Figure 2) (Shin et al., 2018). Color thresholds determined by the Positive Pixel Algorithm (Leica Biosystems, Wetzlar, Germany) were verified by a board-certified pathologist. In total, 22 thrombus samples (one from each patient) were used in this study. Three primary thrombus subgroups were identified based on assessment of the composition: 1) Fibrin-dominant, characterized as being composed of more than 75% fibrin (*N* = 7), 2) Red blood cell-dominant, characterized as being composed of more than 75% red blood cells (*N* = 7), and 3) Half-Half, or thrombi that contained between 25% and 75% red blood cells/fibrin (*N* = 8).

**TABLE 1:** Demographics for volunteer mechanical thrombectomy patients considered in this study.

Patient age (y)			
Average		Range	
57.5		27–85	
Race			
White	Black		Hispanic
6	15		1
Sex			
Male		Female	
8		14	

Venous thrombi were collected from these patients and subjected to colorimetric analysis to identify red blood cell and fibrin composition.

### Pressure distribution of histotripsy source

The degree and type of bubble activity generated depends on the acoustic pressure field of the focused source (Rosnitskiy et al., 2016). These calculations used the measured acoustic field of an eight-element focused transducer designed specifically to treat iliofemoral thrombi (Maxwell et al., 2022). The transducer had an elliptical geometry (7 and 9 cm for the minor and major axes, respectively) and 1.5-MHz fundamental frequency. The pressure distribution measured with a needle hydrophone (HNP-0400, Onda Corp., Sunnyvale, CA, United States) was found to have a focal zone with −6 dB widths of 4.3 mm × 0.7 mm × 0.7 mm along the central axis, and major and minor axes of the source, respectively.

### Monte Carlo calculation to determine locations of bubble nucleation

Bubble nucleation locations were assigned in the calculation using a Monte Carlo approach. A previous study verified this approach provides an accurate assessment of the bubble extent *in vitro* (Maxwell et al., 2013). The calculation is based on *in vitro* and *ex vivo* studies indicating the likelihood of bubble generation increases with the peak negative pressure for intrinsic-threshold exposure schemes consisting of pulses one to three acoustic cycles in duration applied at a slow rate (<5 Hz) (Herbert et al., 2006). For clot, the 50% probability of bubble generation occurs at a peak negative pressure of 26.8 ± 1.2 MPa (Maxwell et al., 2013). The probability of bubble nucleation for each pixel in the calculation was determined based on the pressure profile of the acoustic source. Further, each pixel was assigned a random number between 0 and 1. Nucleation was designated for pixels that had a greater probability for bubble generation than the random number. The Monte Carlo calculations were performed using a two-dimensional computational grid with pixel dimensions of 7.4 µm × 7.4 µm, similar to the size of a red blood cell (Kinnunen et al., 2011). No significant changes in the outcomes of the Monte Carlo calculation were observed for pixel dimensions ranging from 7.4 µm × 7.4 µm to 100 μm × 100 µm (Supplementary Table S1).

### Analytic calculation of histotripsy bubble size

In pixels assigned to be bubble nucleation locations, the bubble size was determined using an established analytic model for histotripsy excitations (Bader and Holland, 2016; Bader, 2018). Briefly, the bubble diameter was calculated assuming the thrombus behaved as a Kevin-Voight viscoelastic media (Schmitt et al., 2011):

$$R_{\mathrm{MAX}}=\left[R_{0}+\sqrt{\frac{2P_{0}\xi }{9\rho }}\tau \right]{\left[\frac{\xi P_{0}}{3p_{EFF}}+1\right]}^{1/3}$$ (1)

where *P*_0_ is the ambient pressure (0.1 MPa), and ρ is the thrombus density (1,000 kg/m^3^). The initial bubble radius *R*_0_ was set to 2.5 nm based on the estimated size of intrinsic nuclei in soft tissues (Maxwell et al., 2013). The effective pressure (*p*_*EFF*_) acts as a “Blake brake” to arrest bubble growth, and depends on the histotripsy insonation scheme (Bader and Holland, 2016). The Monte Carlo assumes short duration pulses similar to intrinsic threshold histotripsy (Maxwell et al., 2013), and the effective pressure can be expressed as (Bader, 2018):

$$p_{EFF}=P_{0}\left(1+4G/3\;P_{0}\right)$$ (2)

where *G* is the elastic modulus. The variable ξ = *ξ*_*HA*_−6*G*/*P*_0_, where *ξ*_*HA*_ is as defined in Holland and Apfel (Holland and Apfel, 1989). Predictions of bubble growth based on Eq. 1 have been shown to be in good agreement with numerical simulations (Bader and Holland, 2016) and within the variability of experimental measurements of bubble size using high speed videography for histotripsy pulses with excitation frequencies ranging from 345 kHz to 3 MHz, and material elastic moduli ranging from 0 kPa (i.e., water) to 600 kPa (Vlaisavljevich et al., 2015; Bader, 2018).

### Incorporation of histology into Monte Carlo simulations

Calculations of the maximum bubble size using Eq. 1 assume a uniform background. Thrombi are spatially heterogenous (Figure 1), and the degree of bubble expansion will differ significantly in fibrin compared to red blood cells (Bader, 2018). To account for the more complex bubble nuclei dynamics in thrombi, pixels in the two-dimensional Monte Carlo calculation were assigned to be fibrin or red blood cells based on the 22 annotated histological sections of *ex vivo* venous specimen. The elastic modulus for fibrin pixels was assigned 14 MPa (Litvinov and Weisel, 2017), and 4 kPa for red blood cell regions (Hendley et al., 2019). To determine the maximum bubble size via Eq. 1, *G* in Eq. 2 was assigned to be the average elastic modulus in the computational grid over a 500 µm radius from the nucleation location. Minimal changes were observed in outcomes for the calculation if the average *G* was calculated based on an area with radius 100–1,000 µm.

### Outcomes for Monte Carlo simulations

**FIGURE 3:**
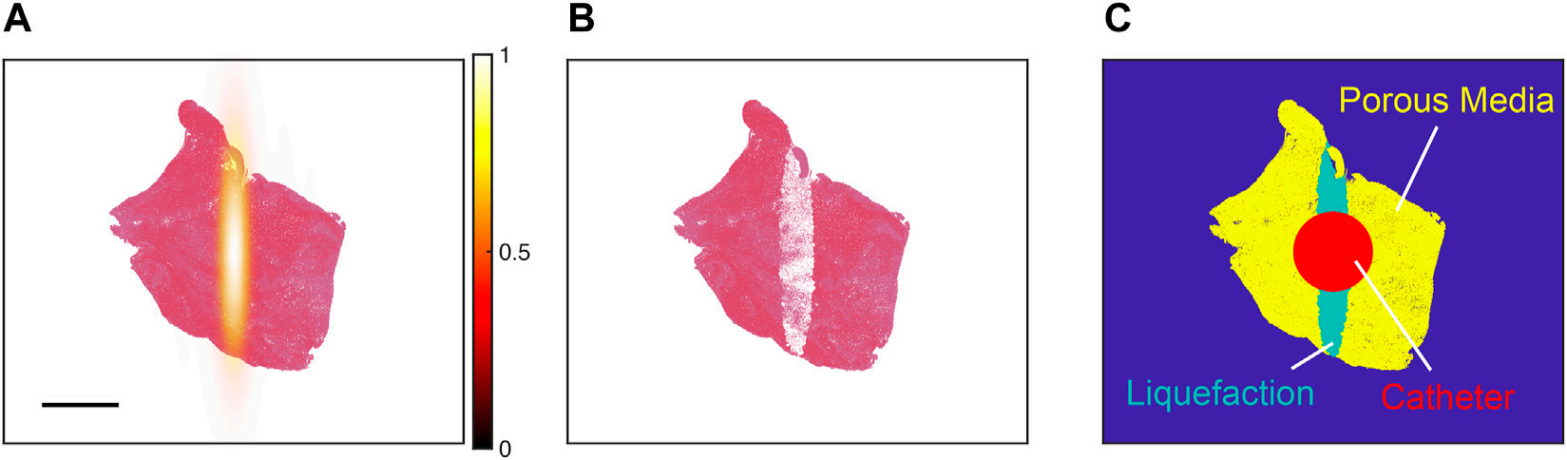
**(A)** Pressure distribution of the focused source (hot colormap) aligned with the centroid of a representative histology thrombus section (red = red blood cells, pink = fibrin). The color bar denotes the normalized peak negative pressure, and the scale bar denotes a 2 mm distance. **(B)** Representative outcome for Monte Carlo calculation. Red blood cell pixels within regions of bubble expansion were assigned to be “ablated” (white pixels). **(C)** For simulations of thrombolytic flow from an infusion catheter, ablated pixels were modeled as liquefaction (i.e., fluid). Viable portions of the thrombus were modeled as a porous media. The catheter served as a boundary condition in calculations of the perfusion-diffusion equation, with fluid flow of thrombolytic (700 nMol) from the catheter at a rate of 0.87 cm/s.

For each histology section, Monte Carlo calculations were performed with histotripsy pulse peak negative pressures between 28 and 40 MPa. These pulse peak negative pressures are consistent with pre-clinical studies that use intrinsic-threshold histotripsy for the treatment of venous thrombosis with no observed effects of pre-focal bubble activity either *in vitro* (Hendley et al., 2022; Maxwell et al., 2022) or *in vivo* (Zhang et al., 2017; Goudot et al., 2020). The calculation was repeated 1,000 times to mimic treatment of the thrombus with multiple pulses. After each iteration of the calculation, pixels classified as red blood cells within the area encompassed by bubbles were re-classified as “liquified” or ablated (Figure 3). To understand the variability for a given set of conditions, the Monte Carlo calculation was performed ten times.

The pixel dimensions correspond that of a red blood cell (∼7.4 µm). “Ablated” red blood cell pixels were tabulated, and the total amount of hemoglobin released was calculated [∼0.03 ng per “ablated” red blood cell pixel (Yoshida et al., 2019)]. Insertion of the infusion catheter into the clot is also known to cause hemolysis (Hendley et al., 2022), and was estimated based on the number of red blood cell pixels that intersected within 0.84 mm of the thrombus centroid (Figure 3). This circular area is equivalent to the cross section of a Cragg-McNamara valued infusion catheter (Medtronic, Minneapolis, MN, United States) commonly used for the treatment of deep vein thrombosis (Hendley et al., 2022).

### Calculation of catheter-directed infusion of thrombolytic

Thrombolytic drug is infused directly into a venous thrombus with an infusion catheter (Chen et al., 2016). Given information about the infusion parameters and modeling the thrombus as a porous media, the distribution of thrombolytic can be determined by solving the perfusion-diffusion equation (Ivanchenko et al., 2012):

$$\frac{\partial C}{\partial t}=A_{1}\frac{\partial^{2}C}{\partial r^{2}}+\frac{A_{2}}{r^{2}}\frac{\partial^{2}C}{\partial \theta^{2}}+\frac{A_{3}}{r}\frac{\partial C}{\partial r}-k_{2}C_{p}C$$ (3)

Here, *C* is the concentration of the thrombolytic drug recombinant tissue plasminogen activator (rt-PA) as a function of the polar coordinates *r* (radial distance) and θ (azimuthal angle), and time *t*. The last term on the right side of Eq. 3 accounts for quenching of rt-PA through its interaction with plasminogen activator inhibitor one (PAI-1) (Binder et al., 2002). The quenching rate is proportional to the concentration of PAI-1 (*C*_*p*_) and the interaction rate *k*_2_ (29 μM^−1^s^−1^) (Shaw et al., 2007).

The constants *A*_1_, *A*_2_, and *A*_3_ reflect the diffusive properties of the thrombus. Prior studies have demonstrated clot diffusivity is increased due to histotripsy ablation (Allen et al., 2017; Anthony et al., 2019). The Monte Carlo calculation was used to inform locations of viable and ablated thrombus when solving Eq. 3, as indicated in Figure 3. Portions of the computation grid associated with viable thrombus (i.e., regions with no bubble activity) estimated *A*_1_, *A*2, and *A*_3_ assuming a porous media as (Bear, 1972):

$$A_{1}=Va_{I}$$ (4)

$$A_{2}=Va_{II}$$ (5)

$$A_{3}=V$$ (6)

Here, V=Q/2πBnr , where *Q* was the discharge rate of thrombolytic from a Cragg-McNamara infusion catheter (Medtronic, Minneapolis, MN, United States). The source boundary conditions were set to be constant over an circular area with radius of 0.84 mm (i.e., radius of Cragg-McNamara catheter) aligned with the centroid of the thrombus (Figure 3). Based on the typical infusion rate of 12.5 mL/h (rt-PA delivery of 0.5 mg/h) (Vedantham et al., 2017) and catheter size, Q ∼ 0.0035 mL/s. The constant *B* is the average distance between the catheter and the edge of the thrombus, and *n* is the thrombus porosity taken here to be 0.15 (Xu et al., 2010). The constants *a*_I_ and *a*_II_ were the longitudinal and transverse dispersity, respectively, which can be estimated based on the Pèclet number (∼232) as (Delgado, 2007):

$$a_{I}=\frac{1}{2}d^{1.2}{\left(\frac{U}{nD_{clot}}\right)}^{0.2}$$ (7)

$$a_{II}=0.025d^{1.1}{\left(\frac{U}{nD_{clot}}\right)}^{1.1}$$ (8)

where *U* is the fluid velocity at the catheter surface (∼0.87 cm/s), and *D*_*clot*_ is the diffusion coefficient for intact thrombus [2.5 × 10^−6^ cm^2^/s (Allen et al., 2017)]. The parameter *d* is the diameter of particulates within the thrombus, which was estimated as a weighted average of the diameter of fibrin bundles [∼10^-5^ cm (Weisel, 2007)] and red blood cells (∼10^-4^ cm). Less than a 5% difference was noted in these calculations for the parameter *d* over the range 10^−4^–10^−5^ cm. Combining these constants together, *a*_*I*_ ∼ 9.4 × 10^−6^ cm^2^/s, and *a*_*II*_ ∼ 3.4 × 10^−6^ cm^2^/s.

Pixels associated with lysed red blood cells in the Monte Carlo calculation were assumed to be fluid (i.e., liquefied), and the constants *A*_1_, *A*_2_, and *A*_3_ in Eq. 3 were estimated as (Ivanchenko et al., 2012):

$$A_{1}=D_{ablate}$$ (9)

$$A_{2}=D_{ablate}$$ (10)

$$A_{3}=D_{ablate}-U$$ (11)

were *D*_*ablate*_ was the diffusion coefficient of ablated thrombus taken to be 25 × 10^−6^ cm^2^/s (Allen et al., 2017).

Equation 3 was solved using an explicit time-marching finite difference time domain scheme (Bouchoux et al., 2012). In order to meet the Courant-Friedrichs-Lewy stability condition (Courant et al., 1928; Sun and Trueman, 2004), the radial, azimuthal, and temporal step sizes were set to 0.001 mm, π/25 radian, and 0.1 ms, respectively. Estimates for fluid flow in the absence of PAI-1 (last term in Eq. 3) were found to be in good agreement with benchmark analytic solutions for flow through porous media (Supplementary Figure S3). Initial calculations were performed out to 20 min, though little change was observed in the distribution of rt-PA after ∼10 s when PAI-1 was included in the calculation (Supplementary Figure S4).

### Assessment of fibrinolysis

Once the distribution of rt-PA was determined via Eq. 3, the concentration of fibrin degradation products (*C*_*FDP*_) was determined for fibrin pixels in the computational grid using the known reaction rate between rt-PA and fibrin (Shaw et al., 2007):

$$C_{FDP}=C_{FIB}\left[1-\exp\left\{-\left(\frac{k_{1}k_{4}CC_{Pm}}{k_{3}C_{AP}}\right)t\right\}\right]$$ (12)

where *C* is the concentration of rt-PA, *C*_*FIB*_ is the initial concentration of fibrin (∼950 nMol for fibrin pixels, 0 for red blood cell pixels), *C*_*Pm*_ is the concentration of plasminogen (0.13 µMol), *C*_*AP*_ is the concentration of α_2_ antiplasmin (0.44 µMol), *k*_1_ is the rate constant for the conversion of plasminogen to plasmin under the action of rt-PA (0.011 µM^−1^s^−1^), *k*_4_ is the rate constant for the conversion of fibrin to fibrin degradation products under the action of plasmin (0.77 µM^−1^s^−1^), and *k*_3_ is reaction rate for quenching plasmin under the action of α_2_ antiplasmin (10 μM^−1^s^−1^) (Shaw et al., 2007). The calculation was performed for *t* = 20 min, similar to that for pre-clinical studies testing histotripsy clot ablation (Maxwell et al., 2011; Goudot et al., 2020; Hendley et al., 2022). The total amount of fibrin degradation products were tabulated for each set of conditions.

### Statistical analysis

Difference in the calculated amount of hemoglobin (i.e., histotripsy ablation) between arms (i.e., histotripsy pulse pressure, thrombus subtype) were determined with ANOVA using Tukey’s HSD to determine significant differences between arms (*p* < 0.05). A similar ANOVA analysis was used to determine the differences between arms for the total fibrin degradation production. For each individual thrombus section, Linear Regression Analysis was used to correlate hemoglobin and fibrin degradation products over the range of histotripsy pulse pressures investigated (28–40 MPa peak negative pressure). Briefly, Z-scores were computed for hemoglobin and fibrin degradation products, and fit in the least-squared sense to a linear polynomial. The slope was the polynomial was used to report the Linear Regression Coefficient. Variations in the Linear Regression Coefficient from one indicate histotripsy preferentially promotes hemolysis or fibrinolysis.

## Results

### Monte Carlo simulations

**FIGURE 4:**
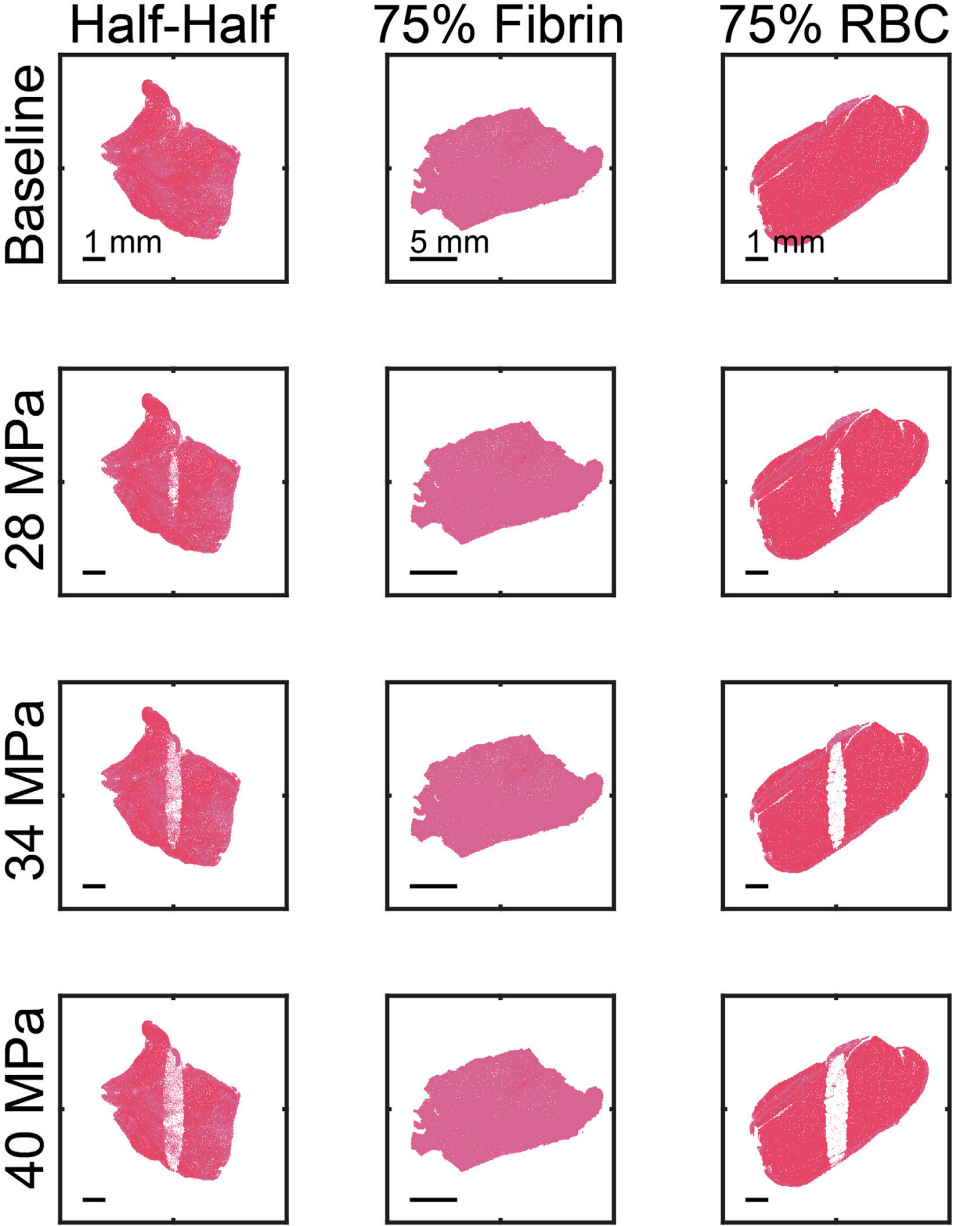
Representative examples of calculated ablation zones (1,000 pulses). The spatial peak, temporal peak negative pressure of the histotripsy pulse is noted along the left of each row. The ultrasound pulse propagation is from top to bottom in the image. The thrombus subgroup is noted at the top of each column.

**FIGURE 5:**
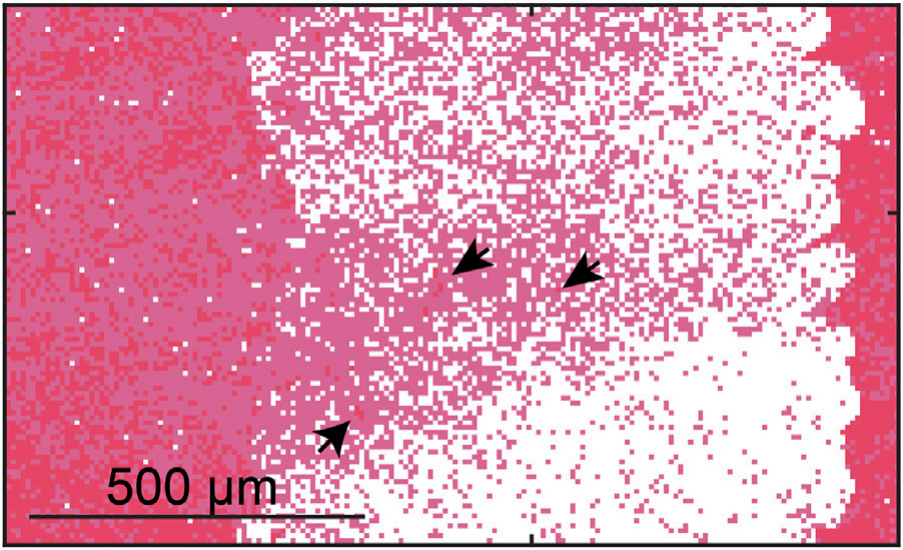
Calculated ablation zone (white) for Half-Half thrombus using a 40 MPa peak negative pressure. Red pixels correspond to red blood cells, and pink pixels correspond to fibrin. Arrows indicate “viable” red blood cells surrounded by fibrin within the focal zone.

Outcomes from the Monte Carlo calculation represent anticipated changes in thrombus structure due to treatment from histotripsy alone. Examples of the Monte Carlo calculation are shown in Figure 4 for each thrombus subgroup. Histotripsy produced no noticeable change in fibrin-dominant thrombi (>75% fibrin). In contrast, minimal residual structure was present within the focal zone for the red blood cell-dominant thrombi (>75% red blood cells). The ablation zone for Half-Half thrombi (between 25% and 75% red blood cells) was disjointed, and regions of fibrin were present within the focal area. Red blood cell clusters were found interspersed within the fibrin-rich regions for Half-Half thrombi (Figure 5) due to the spatial variability of bubble dynamics based on the thrombus heterogeneity.

**FIGURE 6:**
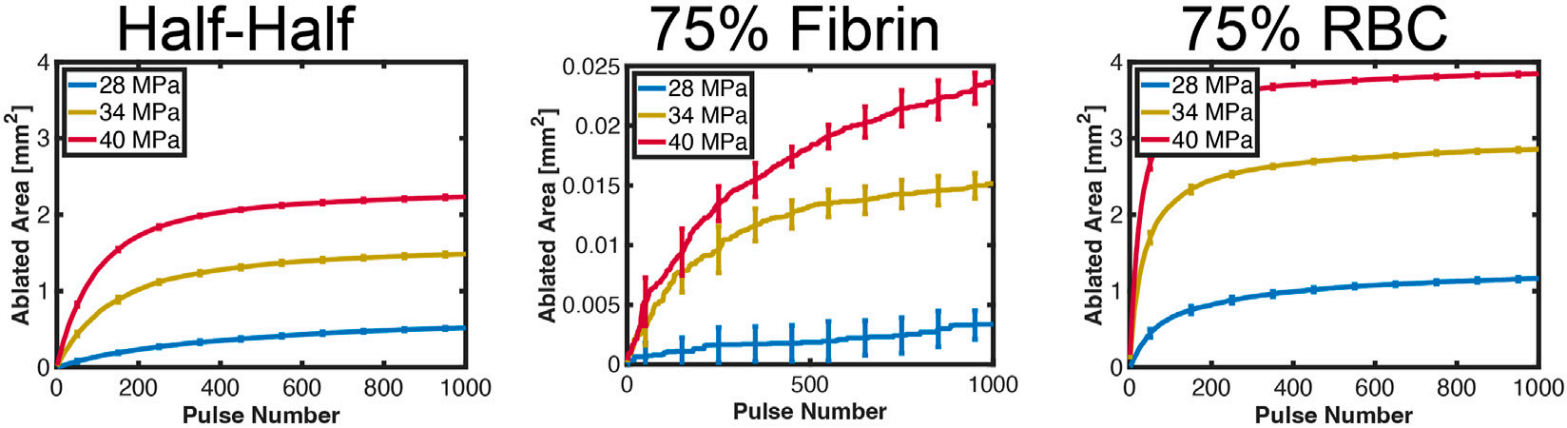
Computed ablation area for each representative examples in each thrombus subgroup. The pulse peak negative pressure is indicated in the legend. The error bars represent the variation for ten independent Monte Carlo calculations (*N* = 10 independent simulations).

The relationship between the ablation area and the number of applied pulses is shown in Figure 6 for representative examples in each thrombus subgroup. The ablation area continually increased with the number of applied pulses for fibrin-dominant thrombi, but was more than one hundred times smaller than the other subgroups. For the other thrombus subgroups, a rapid increase was observed in the ablation area for the first ∼50 (red blood cell-dominant) to 200 (Half-Half) pulses. There was a substantial reduction in the growth of the ablation zone for the application of additional pulses, which depended on the pulse peak negative pressure and thrombus composition. The positive correlation between ablation area and peak negative pressure was the result of an increase in the likelihood of bubble nucleation and bubble expansion.

**FIGURE 7:**
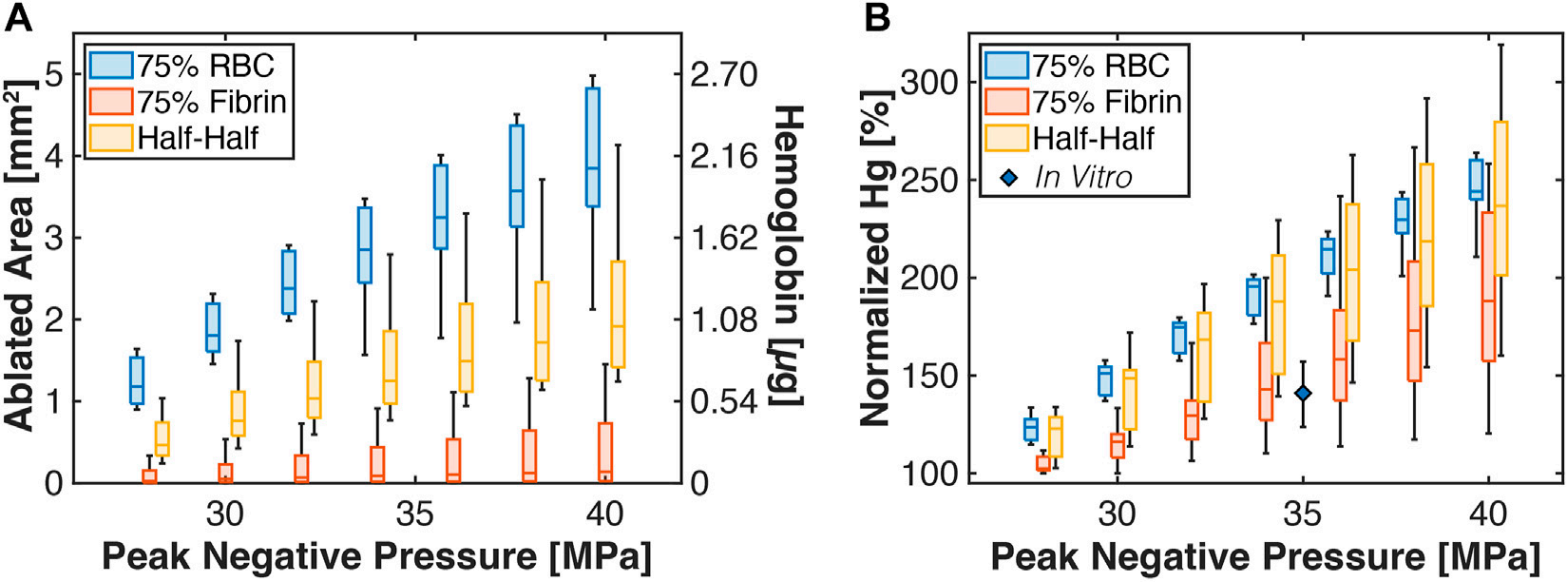
**(A)** Box and whisker plots for the calculated thrombus ablation area (left ordinate) relative to the peak negative pressure of the histotripsy pulse. The right ordinate indicates the calculated amount of hemoglobin generated due to histotripsy exposure. The legend indicates analysis for thrombi subgroups. The ablation area is reported for calculations based on the application of 1,000 pulses. **(B)** Box and whisker plot for the calculated hemoglobin (Hg) generation due to histotripsy bubble activity relative to catheter insertion. The diamond is a measured value for normalized hemoglobin production for clots composed of 80.2% ± 3.3% red blood cell content (Hendley et al., 2022). For all box and whisker plots: The solid line indicates the median value, the box indicates the 25th and 75th percentiles, and the whiskers extend to the most extreme datapoints excluding outliers.

The overall ablation area is noted in Figure 7 relative to the peak negative pressure of the histotripsy pulse (1,000 applied histotripsy pulses). For a given peak negative pressure, red blood cell-dominant thrombi had the largest ablation area relative to the other subgroups, followed by Half-Half. and fibrin-dominant thrombi. The Pearson correlation coefficients between ablation area and the histotripsy pulse peak negative pressure were significant for all groups (*p* < 0.05), but were reduced for fibrin-dominant thrombi (*ρ* = 0.36, *N* = 7) relative to the other subgroups (Half-Half thrombi: *ρ* = 0.64, red blood cell-dominant thrombi: *ρ* = 0.81). The lack of influence of the histotripsy pulse peak negative pressure in fibrin-dominant thrombi is also noted in Supplementary Tables S3–S6. The calculated degree of hemoglobin generated due to “ablation” of red blood cells is also indicated in Figure 7.

The degree of hemoglobin generated due to insertion of the infusion catheter into the thrombus was determined as described in the Methods, and compared to outcomes for histotripsy exposure and insertion of the infusion catheter (red and aqua areas, Figure 3C). For red blood cell-dominant thrombi, histotripsy exposure increased hemoglobin generation relative to catheter insertion for all pulse peak negative pressure investigated (Figure 7A). Fibrin-dominant and Half-Half thrombi required a pulse peak negative pressure of 40 and 32 MPa, respectively, to generate significant increases in hemoglobin relative to the infusion catheter (*p* < 0.05). *In vitro* measurements of hemolysis generated for catheter insertion relative to histotripsy exposure and catheter insertion is also indicated in Figure 7 for clots with 80.2% ± 3.3% red blood cell content (Hendley et al., 2022). The experimental data were found to be in agree with the Monte Carlo calculations for Fibrin-dominant and Half-Half thrombi, but had a lower normalized hemoglobin for red blood cell-dominant thrombi.

### FDTD calculations of rt-PA distribution

**FIGURE 8:**
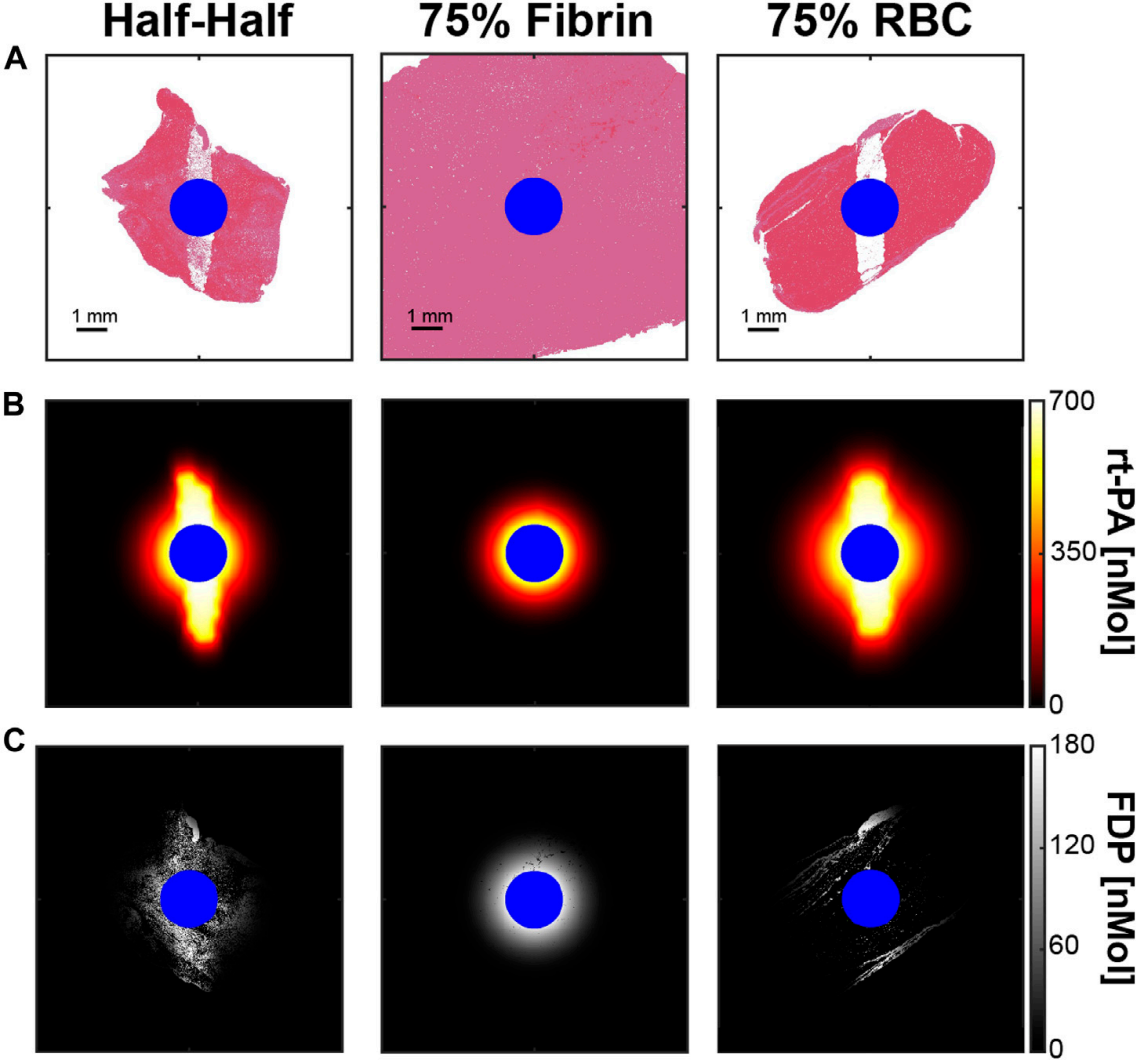
**(A)** Thrombus after the application of 1,000 histotripsy pulses with a peak negative pressure of 40 MPa for each thrombus subgroup. **(B)** FDTD calculation for the steady state distribution of rt-PA within ablated thrombus. **(C)** Calculated concentration of fibrin degradation products (FDP) resulting from a 20 min rt-PA exposure. Blue pixels correspond to the infusion catheter. Columns correspond to thrombus subgroups.

Representative examples for the steady-state distribution of thrombolytic drug based on the FDTD calculation are shown in Figure 8. The concentration of rt-PA and fibrin degradation products was symmetrically distributed around the catheter to a distance of ∼2 mm for fibrin-dominant thrombi, similar to for calculations that did not include the effects of histotripsy (Supplementary Figure S5). For the other thrombus subgroups, asymmetric regions with high concentrations of rt-PA were observed within the histotripsy focal zone. The distribution of fibrin degradation products differed between Half-Half and red blood cell-dominant thrombi, primarily due to the variation in fibrin concentration between the two subgroups.

**FIGURE 9:**
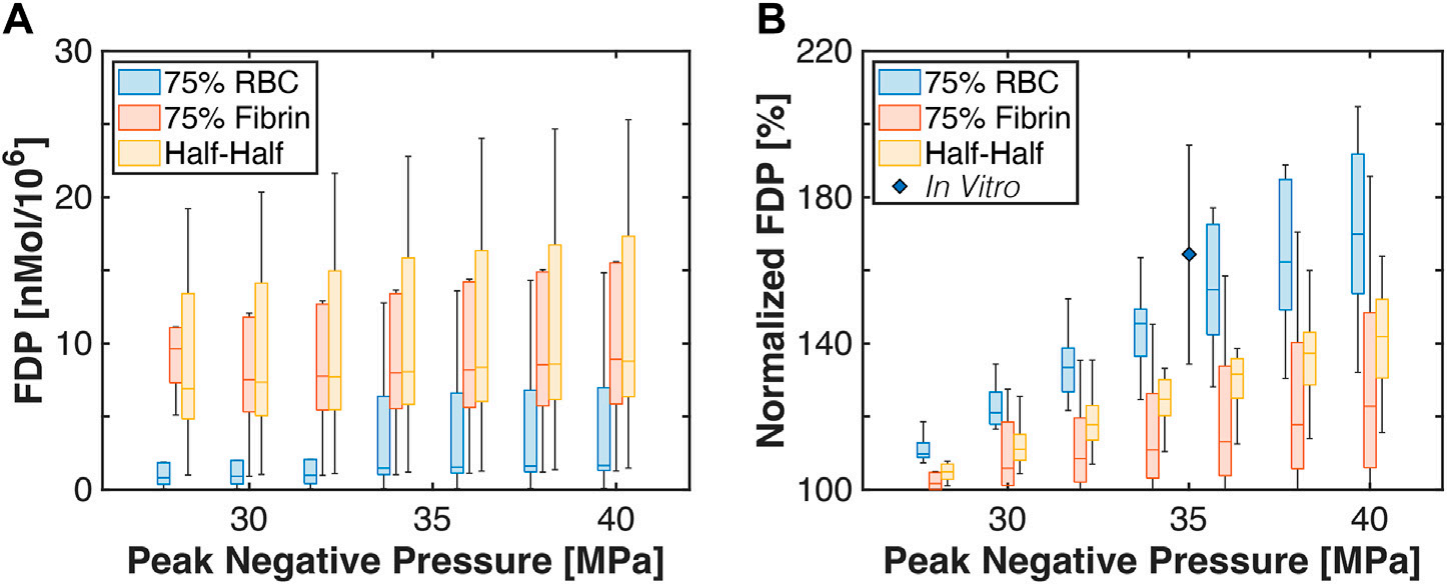
**(A)** Box and whisker plots for the computed amount of fibrin degredation product generated. Calculations were performed for the application of 1,000 histotripsy pulses. **(B)** Box and whisker plot for the computed fibrin degredation products relative to baseline (i.e., no histotripsy added) for each thrombus subgroup. The diamond indicates measurements for prior studies conducted *in vitro* with red blood cell-rich clots (Hendley et al., 2022). For box and whisker plots, the solid line indicates the median value, the box indicates the 25th and 75th percentiles, and the whiskers extend to the most extreme datapoints excluding outliers.

The total amount of fibrin degradation products generated is shown in Figure 9. The histotripsy pulse peak negative pressure had no observed influence on the computed fibrinolysis calculation, potentially due to variability in fibrin concentration even within thrombus subgroups. For a given thrombus, a relative fibrin degradation product value was computed as:

$$\frac{FPD_{PNP}}{FDP_{0\;MPa}}$$ (13)

where *FDP* is the fibrin degradation product total, and the subscript denotes the peak negative pressure (*PNP*) (i.e., 0 MPa indicates no histotripsy). For a given histotripsy pulse peak negative pressure, red blood cell-dominant thrombi had the largest relative fibrin degradation product generation compared to the other subgroups. Half-Half thrombi had an increased relative fibrin degradation product generation compared to the fibrin-dominant subgroup for peak negative pressures between 32 and 38 MPa, but equivalent at lower and high histotripsy exposure conditions. An *in vitro* measurement of relative fibrin degradation product generation for clots (80.2% ± 3.3% red blood cell content) is also indicated in Figure 9 (Hendley et al., 2022). Measured values agreed with the calculations for red blood cell-dominant and Half-Half clots within experimental variability, but were increased relative to fibrin-dominant thrombi.

Change in fibrin degradation product generation with the histotripsy pulse pressure was also found to vary with subgroup. The Pearson’s correlation coefficients were significant for each subgroup: 0.19 fibrin-dominant, 0.42 for Half-Half, and 0.67 for red blood cell-dominant. Comparison in the degree of fibrinolysis between peak negative pressure arms for each thrombus subgroup are reported in Supplementary Tables S7–S10. Each successive pressure level resulted in an increased fibrinolysis for Half-Half thrombi, but larger pressure differences were required for the other thrombus subgroups.

### Connection between hemolysis and fibrinolysis

**FIGURE 10:**
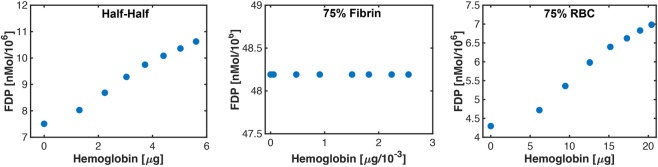
Representative correlations observed between hemolysis (hemoglobin) and fibrin degradation products (FDP) for each thrombus subgroup. The changes in hemoglobin and fibrinolysis occur because of the variability in bubble activity over the range of histotripsy pulse pressures investigated in this study. Error bars are included in each figure to indicate the average of ten independent Monte Carlo calculations, but are smaller than the actual markers.

The correlation between hemolysis and fibrinolysis is shown in Figure 10 for representative specimens in each thrombus subgroup. Changes in hemoglobin or fibrin degradation product occur due to the range of histotripsy pulse peak negative pressures explored in this study. For thrombi composed of more 25% red blood cells (Half-Half or red blood cell-dominant subgroups), positive trends were observed between hemolysis and fibrinolysis. In contrast, no relationship was observed between hemolysis and fibrinolysis for fibrin-dominant thrombi.

**FIGURE 11:**
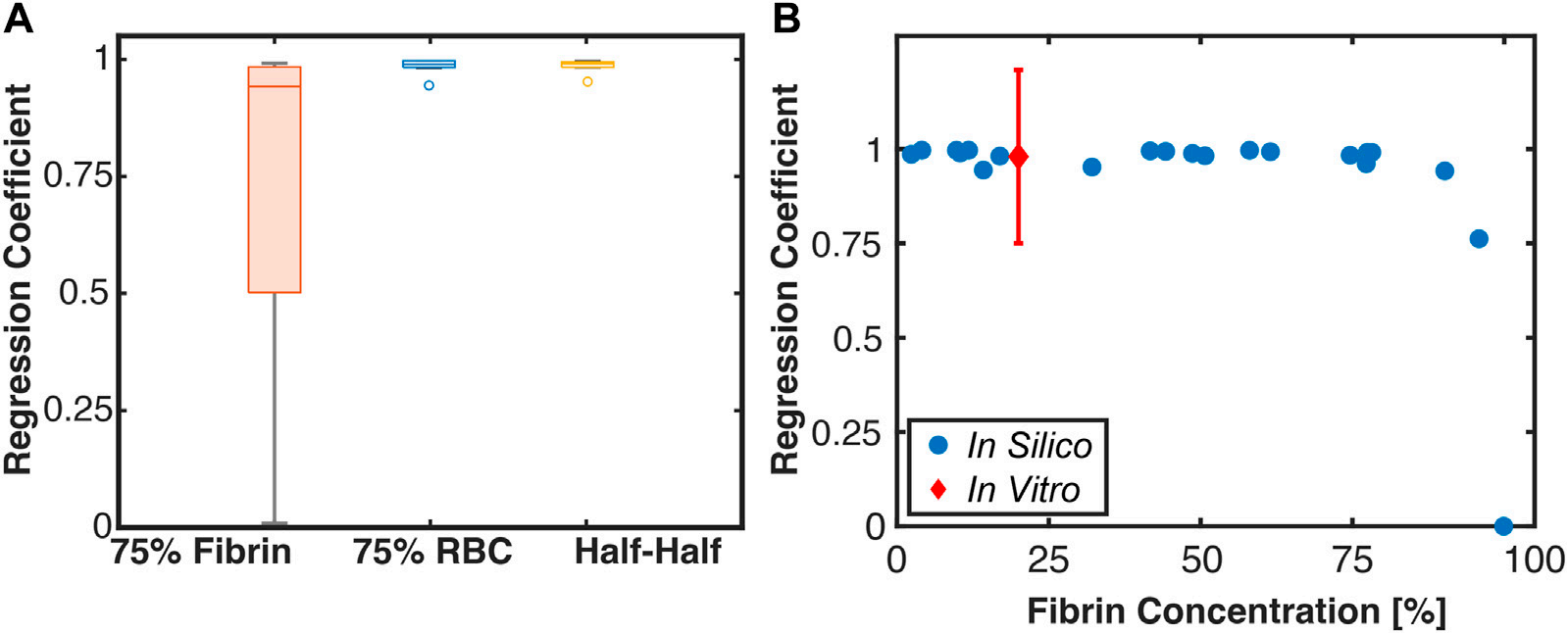
**(A)** Box and whisker plot for the Linear Regression Coefficient between calculations for hemolysis (Monte Carlo calculation) and fibrinolysis (FDTD calculation). The solid line is the median of the data, the box extends to the 25% and 75% percentiles, and whiskers extends to the range of data not considered outliers. The circle markers are considered outliers to the data (*N* = 7 each for 75% fibrin and 75% red blood cell (RBC) dominant thrombi, *N* = 8 for Half-Half). **(B)** Scatter plot of the Linear Regression Coefficient for all tested thrombi relative to the fibrin concentration. The red marker is a measured Linear Regression Coefficient based on *in vitro* data for single-cycle histotripsy pulses (Hendley et al., 2022).

The Linear Regression Coefficients for red blood cell-dominant and Half-Half thrombi were found to be approximately one (Figure 11), indicating hemolysis and FDP increase proportionally as the histotripsy pulse pressure increases. In contrast, fibrin-dominant thrombi exhibited a wide range of Linear Regression Coefficients, which rapidly decreased to zero as the fibrin concentration exceeded ∼95% (Figure 11). The measured Linear Regression Coefficient for red blood cell-dominant clots exposed to histotripsy and rt-PA *in vitro* is also indicated in Figure 11 (Hendley et al., 2021a).

## Discussion

### Histotripsy clot debulking to enhance thrombolytic delivery

The goal of this study was to assess changes in the intra-thrombus delivery profile of thrombolytic drugs via catheter infusion following histotripsy exposure. Thrombus in its native state was modeled as a porous media (Bear, 1972), with regions exposed to histotripsy assumed to have an increased diffusivity (Allen et al., 2017). A key finding for this study was that histotripsy increases the extent of rt-PA distribution, resulting in increased fibrinolysis (Figure 10). These effects depended on the thrombus composition, and were most prominent for subgroups with appreciable red blood cell.

There may be other effects besides changes in thrombus diffusivity that could account for the experimental observation that histotripsy improves rt-PA efficacy (Hendley et al., 2021a). Convective flow due to the simultaneous action of bubble oscillations and drug delivery is a hypothesized mechanism for enhanced efficacy in other ultrasound-mediated therapies (Kooiman et al., 2020; Stride et al., 2020). Histotripsy-induced flow was neglected in these calculations, though outcomes were found to be in good agreement with *in vitro* studies (Figures 9, 11). Thus, the contribution of bubble-induced convective flow appears to be a secondary effect relative to increasing thrombus diffusivity for histotripsy. This observation may be in part because histotripsy bubbles are generated within soft tissues where flow will be significantly restricted compared to targets that include a tissue/fluid interface (Bear, 1972). The ability to apply histotripsy and thrombolytic independent of each other may increase the flexibility of the treatment pipeline and reduce procedural complexity.

Overall, these findings suggest histotripsy promotes intra-thrombus delivery of thrombolytic primary via increasing diffusivity of the target. There are other means to increase tissue diffusivity such as heating, though a prior study found temperature does not improve the action of thrombolytic drug (Nahirnyak et al., 2007). Histotripsy liquefies or disintegrates tissue (Bader et al., 2019), which will provide a more permeant change in diffusivity compared to heating. Venous thrombosis can be 5 cm or longer (Garcia and Labropoulos, 2023), which motivates the development of rapid insonation schemes to accentuate whole-thrombus diffusivity (Zhang et al., 2016). Indeed, a prior study indicated a trend of increasing fibrinolysis for treatment schemes that insonified a ten-fold increase in the cross section of the clot relative to a single target (Hendley et al., 2022). Future calculations will focus on histotripsy exposure schemes to optimize rt-PA enhancement.

### Monte Carlo calculation

The use of segmented histological sections (*N* = 22) in the Monte Carlo calculation provided insights to the anticipated variability in outcomes for application of histotripsy to venous thrombosis. Bubble nucleation was not observed outside the thrombus area (i.e., no prediction of off-target damage), consistent with previous pre-clinical studies (Zhang et al., 2017; Hendley et al., 2021a). Appropriate pulsing conditions should be considered to minimize the potential for pre-focal bubble activity (Zhang et al., 2015). The calculated ablation zones were found to be non-uniform in fibrin-dense regions of the thrombus (Figure 4). This finding is consistent with observations *ex vivo* and *in vivo* that extracellular structures are resistant to treatment by histotripsy (Wang et al., 2012; Wang et al., 2013; Hendley et al., 2021a). The persistent fibrin structure represents residual thrombus not fully treated by histotripsy alone. The rate of re-occlusion is increased if there is residual thrombus in the vessel (Conklin et al., 2009), and therefore increases the need for re-treatment with histotripsy. By combining histotripsy with catheter-directed thrombolytic, both fibrin and cellular components of the thrombus can be addressed (Hendley et al., 2021a).

Viable red blood cells were found interspersed within fibrin-dense regions in the focal zone (Figure 5). Tumor ablation studies with histotripsy indicate cells in close proximity to large vessels composed of extracellular structure are undertreated (Ruger et al., 2023). These findings suggest it is unlikely cells within a stiff microenvironment will be effectively targeted by histotripsy, and necessitate an adjuvant approach. Monte Carlo calculations may help to inform effective dosing strategies provided information on composition, or the likelihood that adjunct treatments (e.g., rt-PA for thrombus, chemotherapy for tumor) will be required to treat residual disease.

*In vitro* measurements of normalized hemoglobin production for red blood cell-dominant clot were consistent with Monte Carlo calculations for Half-Half and fibrin-dominant thrombi, but not red blood cell-dominant thrombi (Figure 7). There are multiple potential reasons for the discrepancy between the *in vitro* measurements and calculations. The Monte Carlo calculations assumes that a new and independent bubble cloud is generated with each histotripsy pulse, which occurs if the pulsing rate is sufficiently slow (<∼5 Hz) (Maxwell et al., 2013). Under these assumptions, good agreement has been found between the predictions of the calculation and the length of the ablation zone (Lin et al., 2014), as indicated in Supplementary Figures S1, S2. The *in vitro* data shown in Figure 7 relied on a 40 Hz pulsing rate (Hendley et al., 2022), which is known to produce a smaller ablation area relative to a 5 Hz rate (Wang et al., 2012). Hence, the Monte Carlo calculations likely overestimate hemoglobin production relative to prior *in vitro* or *in vivo* studies of histotripsy clot ablation.

### Distribution of thrombolytic drug

Several features of interest were generated by the FDTD calculation, even without histotripsy. Thrombolytic drug did not penetrate further than ∼2 mm from the catheter due to the combined effects of the infusion rate and PAI-1 (Supplementary Figure S5), suggesting the standard-of-care generates a narrow channel. There may be modifications to the infusion protocol that can be adopted to improve catheter-directed therapy that do not require histotripsy. A pulsed infusion scheme with high and low flow rates will increase the penetration of rt-PA, and has shown promise in a clinical trial (Elbasty and Metcalf, 2017). Further, concurrent delivery of a PAI-1 inhibitor to mitigate the rapid quench of rt-PA warrants further investigation (Baluta and Vintila, 2015).

### Thrombus subgroup

A major finding in these studies was the influence of thrombus subgroup on outcomes. The thrombus composition is related to disease stage and susceptibility to thrombolytic drugs (Hirsh and Hoak, 1996). Red blood cell-dominant thrombi are primarily acute (<7 days age) (Hendley et al., 2021b), and were the most responsive to the effects of histotripsy in this calculation. There is some question on the benefit of histotripsy for this subgroup given the known efficacy of thrombolytic for acute thrombus. Nevertheless, such an approach may minimize potential off-target effects associated with thrombolytic via increasing the overall efficacy and reducing the procedure duration (Wang et al., 2010). Half-Half thrombi are likely subacute (e.g., greater than 7 days old, less than a month), and are known to be more resistant to thrombolytic than acute thrombosis (Hendley et al., 2022). The residual red blood cell content of subacute thrombi will still be susceptible to histotripsy, which may make subacute thrombus an ideal pathology for histotripsy and thrombolytic.

For Half-Half and red blood cell-dominant thrombus subgroups, hemolysis and fibrinolysis were found to increase in proportion to the histotripsy exposure conditions (i.e., Linear Regression Coefficient ∼1, Figure 11), consistent with findings *in vitro* (Hendley et al., 2021a). As the fibrin concentration increased above ∼95%, only fibrinolysis was observed. Chronic thrombus (i.e., >30 days old) is primarily composed of extracellular structures with minimal red blood cells (Hendley et al., 2021b). Such chronic thrombus may require advances in histotripsy treatment schemes. Recent studies have successfully applied histotripsy to fibrotic prostate (Singh et al., 2022) and tendons (Smallcomb et al., 2021), indicating further investigation is needed to assess its role for chronic venous thrombosis.

The histotripsy insonation scheme necessary to enhance fibrinolysis depended on the thrombus subgroup. The pulse peak negative pressure required to increase fibrin degradation product generation relative to thrombolytic alone scaled with the concentration of fibrin: 28, 30, and 36 MPa for the red blood cell-dominant, Half-Half, and fibrin-dominant thrombus subgroups, respectively (Figure 9). Fibrin degradation was promoted at these pulse peak negative pressures was due to an increased distribution of thrombolytic drug within ablated areas (Figure 8). The number of pulses required to maximize the ablation area for each respective thrombus subgroup was found to be 160 ± 52 pulses for red blood cell-dominant, 200 ± 63 pulses for Half-Half, and 390 ± 76 pulses for fibrin-dominant (Supplementary Table S11). Overall, these data indicate histotripsy fewer pulses with lower peak negative pressures are required to enhance fibrinolysis as the concentration of fibrin decreases.

### Limitations

There are several limitations to this study that prohibit generalizability to the findings. Our calculations assumed the fibrin mesh was unchanged after histotripsy exposure. This assumption is consistent with prior studies indicating histotripsy alone generates no fibrin degradation products (Hendley et al., 2021a; Hendley et al., 2022). There may be changes in the fibrin stiffness not accounted for here that influence the bubble dynamics. Thrombi were assumed to be composed of only fibrin and red blood cells. Additional components are also known to be present within venous thrombi, including increased extracellular collagen structure for chronic disease (Hendley et al., 2021b). The catheter was assumed to be positioned at the center of the thrombus, whereas the precise position will vary based on tissue structure. Interactions between the histotripsy bubble cloud and the catheter were not considered in the model. A previous study demonstrated there appeared to be little change in the bubble cloud dynamics in the presence of an infusion catheter (Hendley et al., 2022). Further, the catheter did not appear to be damaged by histotripsy exposure. A single focal insonation was considered in these studies. Exposure schemes that scan the focal volume laterally within the thrombus have been shown to improve outcomes (Zhang et al., 2016; Hendley et al., 2022), and will be the focus of future calculations. A single focal insonation through the center of the thrombus minimizes the potential for damage to the vein, and would be a likely initial insonation scheme in future clinical trials. The Monte Carlo calculation assumes focused ultrasound pulses of short duration (less than three cycles) are applied at a slow rate (<5 Hz) for bubble nucleation (Maxwell et al., 2013). Calculations in this study were compared to *in vitro* data that included a pulsing rate of 40 Hz and pulse durations ranging from one to twenty acoustic cycles (Hendley et al., 2021a; Hendley et al., 2022). Therefore, the Monte Carlo calculation likely oversimplifies the bubble dynamics relative to the *in vitro* studies. The extension of these calculations to other insonation schemes in heterogenous thrombus will be a focus of future studies. The contribution of thrombus heating from the histotripsy excitation was not included. Changes in tissue temperatures are largely negligible for the short-duration excitations considered in this study (Khokhlova et al., 2015). This study used a two-dimensional computational grid to accommodate the information of thrombus composition based on the histological section. In practice a volume of thrombus will be exposed to histotripsy bubble activity. Extension of the Monte Carlo calculation to three dimensions indicated the ablation zone to be consistent with the focal dimensions of the acoustic source in homogenous media (Supplementary Table S2). Future studies will consider outcomes for histotripsy in a heterogenous volumetric environment. The effects of the endogenous tissue plasminogen activator was not considered in this study. Plasma levels of endogenous fibrinolytic factors were estimated to generate ∼ one percent the fibrin degradation products relative to catheter-directed therapies due to their reduced prevalence (Essig et al., 2021). Overall, the findings of this study suggest increases in thrombus diffusivity due to histotripsy exposure provides an avenue to improve catheter-directed delivery of thrombolytics.

## Data Availability

The raw data supporting the conclusion of this article will be made available by the authors, without undue reservation.
