# Supplementary material for: In silico assessment of histotripsy-induced changes in catheter-directed thrombolytic delivery
Source: Front Physiol. 2023 Jun 28;14:1225804. doi: 10.3389/fphys.2023.1225804 (PMC10336328; doi:10.3389/fphys.2023.1225804)
Supplement: Supplementary file 1 [file DataSheet1.docx]

**Supplementary Information:**

**Predictions of changes to clot structure due to histotripsy and thrombolytic therapy**

Assess Influence of Pixel Size on Monte Carlo Simulation of Ablation Area

The Monte Carlo calculation was performed over a range of computation grid sizes (7.4 x 7.4 µm to 500 x 500 µm) to assess the role of pixel size in estimating the ablation area. The smallest pixel size corresponded to the diameter of a erythrocyte (1), and were used in calculations for results reported in the manuscript. The upper pixel length is consistent with hydrophone spacing when measuring the field of a focused source (2). Simulations were conducted assuming a uniform medium with bulk tissue properties of clot (2), and the acoustic field was as described in the Methods section of the text. The ablation areas for the application of 1,000 pulses are reported in **Table S1** relative to the peak negative pressure of the histotripsy pulse. The mean and standard deviation are reported for ten independent calculations of the Monte Carlo simulation. For all exposure conditions, no differences were noted in the ablation area if the pixel dimension was less than 150 µm x 150 µm. The ablation area decreased for larger pixel dimensions.

| \| PNP [MPa]/ Dimension [µm] \| 7.4 \| 22.1 \| 51.5 \| 110.3 \| 150 \| 250 \| 500 \| \| --- \| --- \| --- \| --- \| --- \| --- \| --- \| --- \| \| 28 \| 2.30 ± 0.04 \| 2.27 ± 0.07 \| 2.22 ± 0.05 \| 2.16 ± 0.07 \| 2.30 ± 0.03 \| 1.50 ± 0.08 \| 1.55 ± 0.11 \| \| 34 \| 4.51 ± 0.03 \| 4.47 ± 0.03 \| 4.48 ± 0.03 \| 4.32 ± 0.03 \| 4.60 ± 0.03 \| 3.3 ± 0.05 \| 2.55 ± 0.11 \| \| 40 \| 6.20 ± 0.05 \| 6.14 ± 0.03 \| 6.14 ± 0.03 \| 6.00 ± 0.03 \| 6.12 ± 0.03 \| 4.60 ± 0.09 \| 4.70 ± 0.20 \| |
| --- | --- | --- | --- | --- | --- | --- | --- | --- | --- | --- | --- | --- | --- | --- | --- | --- | --- | --- | --- | --- | --- | --- | --- | --- | --- | --- | --- | --- | --- | --- | --- | --- |
| **Table S1**. Predicted ablation area with the Monte Carlo simulations of bubble nucleation in units of µm^2^ as a function of the pixel dimension and the histotripsy pulse peak negative pressure (PNP). Values are reported as the mean ± standard deviation for ten independent calculations for each condition. Note that changes in the estimated ablation area are within 5% for pixel dimensions less than 150 µm (*p* < 0.05). Results reported in the main text used a pixel dimension of 7.4 µm, consistent with the diameter of a erythrocyte. |

Assessment of Ablation Volume with Monte Carlo

To incorporate information of the histology stains of *ex vivo* venous thrombi, Monte Carlo calculation in the main text reported outcomes in two dimensions. During benchtop and *in vivo* studies, ablation will occur over a volume. Tissue ablation will occur predominately along the axial dimension (i.e., the acoustic axis), with the lateral and elevational dimensions reduced by nearly a factor of ten (3). To determine any limitations in terms of estimating the volume of ablation with the model used here, Monte Carlo calculations were conducted in three dimensions using a beam profile that was identical along the elevational/lateral dimensions. The calculations were conducted using bulk tissue properties for clot (2). Regions of ablation were predicted with 1,000 iterations of the Monte Carlo calculation using pulses with peak negative pressures between 28 and 40 MPa.

The ablation area in the axial/lateral plane and axial/elevational planes were fit to an elliptical shape to estimate the semi-major (*a*) and semi-minor (*b*) axes. The ablated volume was then reported for each plane as $\text{4π}\text{a}\text{b}^{\text{2}}\text{/3}$, as indicated in **Table S2**. No significant differences were noted between the two reported ablation volumes (*p* > 0.05). Reported outcomes in the primary text restrict analysis to two dimensions, which means there is some loss of information regarding the ablation volume along the elevational dimension. The data in **Table S2** indicate the overall volume of ablation can be estimated given the known beam profile of the acoustic source. A primary concern for the study here were changes in the bubble dynamics due to thrombus inhomogeneities. Volumetric inhomogeneities could not be assessed in three dimensions due to the two-dimensional nature of the histological stains. Future work will incorporate volumetric imaging sensitive to thrombus components, such as Quantitative Susceptibility Mapping with MRI (4).

| \| PNP [MPa]/Volume \| Axial/Lateral [mm^3^] \| Axial/Elevational [mm^3^] \| \| --- \| --- \| --- \| \| 28 \| 1.75 ± 0.15 \| 1.78 ± 0.13 \| \| 34 \| 6.70 ± 0.25 \| 6.73 ± 0.22 \| \| 40 \| 12.66 ± 0.22 \| 12.69 ± 0.23 \| |
| --- | --- | --- | --- | --- | --- | --- | --- | --- | --- | --- | --- | --- |
| **Table S2.** Estimated volume of ablation zone for the application of 1,000 histotripsy pulses at peak negative pressures of 28 to 40 MPa. Estimates of the volume were made assuming the ablation zone was ellipsoidal, and using the semi-major axial distance and either the semi-major lateral distance or semi-major elevational distance of the ablation zone. In all cases, there was no difference in the ablation volume using either the lateral or elevational distance to estimate the volume (*p* > 0.05). Data are reported as the mean ± standard deviation for ten iterations of the Monte Carlo calculation for a given set of conditions. |

Comparison of Monte Carlo Predictions and Experimental Observations:

Red blood cell phantoms were generated following an established protocol (5,6), and exposed to ablative focused ultrasound pulses with a 1-MHz transducer (7). Images of red blood cell disruption were acquired, and compared to predictions of ablation with the Monte Carlo calculation (**Fig. S1**). At all pressures, the length of the ablation area was consistent with predictions of the Monte Carlo calculation (*p* > 0.05 for each pressure level). In contrast, the width of the ablation zone was significantly reduced (*p* < 0.05 based on single-sided t-test for reach pressure level) for the Monte Carlo simulation relative to the in vitro data by ~ 25% (0.6 mm), as indicated in **Fig. S2**. Overall, these calculations indicate the Monte Carlo calculation provide a good estimate of the length of the ablation zone along the central axis of the transducer, but will under predict the ablation width (and therefore overall ablation area).

| 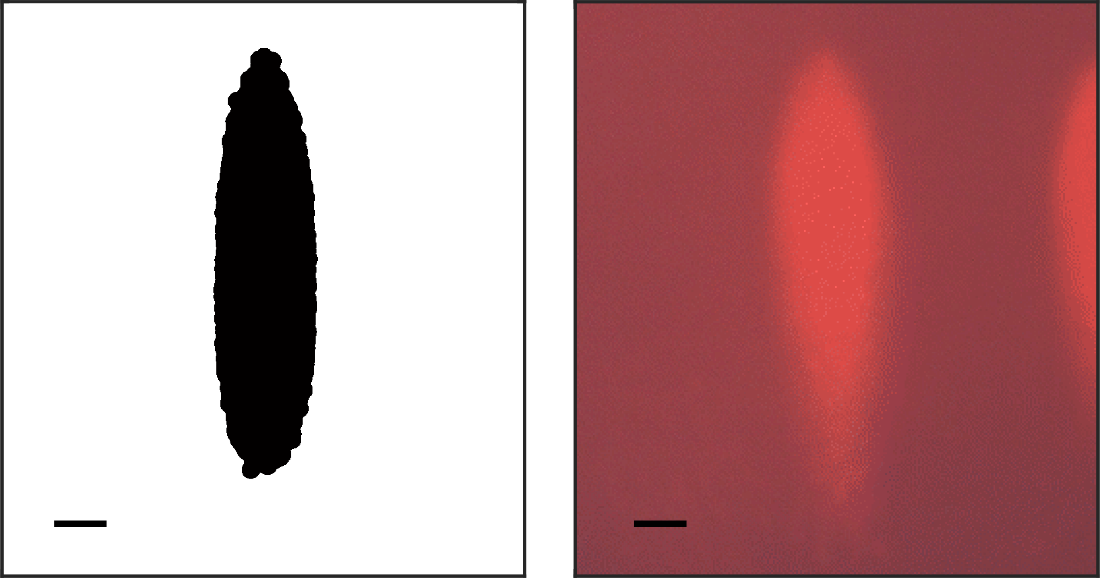 |
| --- |
| **Fig. S1**. Comparison of outcomes for predictions of the ablation area based on the Monte Carlo calculation (left) and that observed in a red blood cell phantom (right). The black bar corresponds to a distance of 1 mm. |
| 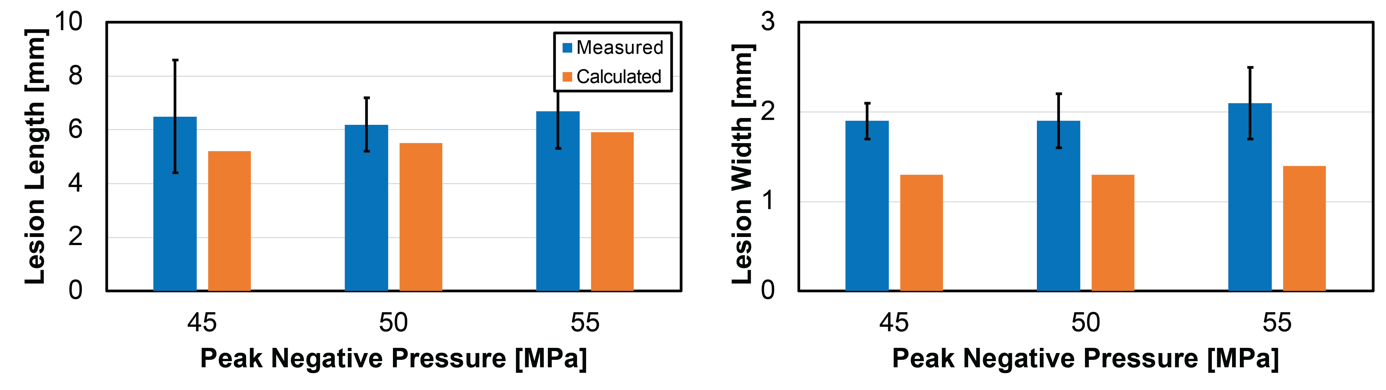 |
| **Fig. S2**. Comparison of outcomes for predictions of the ablation dimensions based on the Monte Carlo calculation. |

Validation of FDTD Solution of the Perfusion/Diffusion Equation:

The finite difference time domain solution of the perfusion-diffusion equation (**Eq. (3)**) was compared to benchmark analytic solutions for forced fluid flow in porous media. For radially symmetric flow from a constant flow source, the concentration of fluid administer from the source can be calculated as (8):

| $\frac{C}{C_{0}}=\frac{1}{2}\mathrm{erf} \left\{ \frac{\frac{r^{2}}{2}-Gt}{\sqrt{\frac{4a_{I}B^{3}}{3}}} \right\}$ | (S1) |
| --- | --- |

where *C*_0_ is the concentration of fluid from the source, *r* is the radial distance from the center of the source, *a_I_* is the longitudinal permittivity, *B* is the distance between the source and the edge of the porous media (here, the porous media is assumed to be cylindrical), and $G=\frac{Q}{2\pi Bn}$, where *Q* is the flow volume rate of fluid from the source, and *n* is the tortuosity of the porous media. Values for these constants are reported in “Calculation of Catheter-Directed Thrombolytic” in the **Methods** Section. It should be noted that the accuracy of **Eq. (S1)** is reduced near the source (i.e., near *r* = 0).

A comparison between predictions the FDTD model and **Eq. (S1)** are shown in **Fig. S3**. Here, damping effects were removed from the FDTD model, which equates to neglecting the term *k*_2_*C_p_C* in **Eq. (3)**. The radial (1 µm) and azimuthal (π/25 radians) step sizes were consistent with that used for the data reported in the **Results** section. Good agreement was found between the FDTD solution and analytic calculation, validating the FDTD model used in this study. These results also highlight the strong influence damping mechanisms (i.e., PAI-1) has on the perfusion of rt-PA into a clot by contrasting the data presented in **Fig. S3** with that of **Fig. 8**.

| 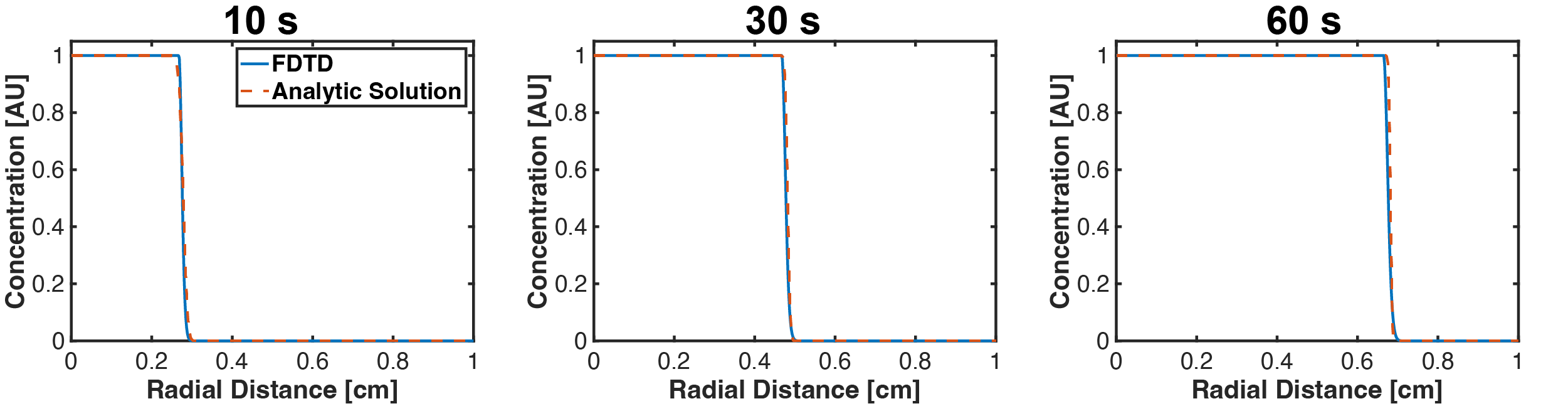 |
| --- |
| **Fig. S3**. Comparison for predictions of fluid flow from a radially symmetric source for finite difference time domain (FDTD) computations via **Eq. (3)** and an analytic solution via **Eq. (S1)**. Good agreement was observed between the two methods, indicating the FDTD model passed appropriate benchmarks. |

Assessment of PAI-1 on rt-PA Distribution:

An example FDTD calculation that incorporates the effects of PAI-1 is shown in **Fig. S4**. Parameters for the calculation are as described in “Calculation of Catheter-Directed Thrombolytic” in the **Methods** Section. In the absence of PAI-1, a distinctive front of thrombolytic is noted (**Fig. S3**). In contrast, little change in the lytic distribution is noted after ~ 10 s of infusion in the presence of PAI-1. Further, rt-PA is completely quenched at distances further than 0.2 cm from the catheter.

|  |
| --- |
| **Fig. S4.** Radial distribution of rt-PA at time points between 0 and 30 seconds. In this simulation, no histotripsy was performed, so the rt-PA distribution was radially symmetric. Timepoints are indicated in the legend. The catheter radius was 0.084 cm. Minimal change in the rt-PA distribution was observed for time points greater than ~ 10 seconds. |

Comparison of Ablation Area Between Histotripsy Pulse Pressures

Following Monte Carlo calculations for the application of 1,000 histotripsy pulses, the ablation area was tabulated for each clot section and each peak negative pressure of the histotripsy pulse. To determine differences in the ablation area between histotripsy pulse peak negative pressures, ANOVA was conducted, using Tukey’s HSD to determine differences between groups (*p* < 0.05). Analysis was conducted for all clots, and each clot subgroup.

| \| **PNP/PNP** \| 30 \| 32 \| 34 \| 36 \| 38 \| 40 \| \| --- \| --- \| --- \| --- \| --- \| --- \| --- \| \| 28 \| **0.68** \| **0.98** \| **1.21** \| **1.45** \| **1.67** \| **1.88** \| \| 30 \|  \| 0.64 \| **0.86** \| **1.11** \| **1.33** \| **1.53** \| \| 32 \|  \|  \| 0.55 \| **0.80** \| **1.02** \| **1.23** \| \| 34 \|  \|  \|  \| 0.58 \| **0.80** \| **1.00** \| \| 36 \|  \|  \|  \|  \| 0.55 \| **0.76** \| \| 38 \|  \|  \|  \|  \|  \| 0.54 \| |
| --- | --- | --- | --- | --- | --- | --- | --- | --- | --- | --- | --- | --- | --- | --- | --- | --- | --- | --- | --- | --- | --- | --- | --- | --- | --- | --- | --- | --- | --- | --- | --- | --- | --- | --- | --- | --- | --- | --- | --- | --- | --- | --- | --- | --- | --- | --- | --- | --- | --- |
| **Table S3.** Differences in the ablation area in millimeters squared between pressure groups when comparing all clots (N = 22 per peak negative pressure). The peak negative pressure (PNP) is reported in units of MPa. Bold values indicate significant differences between the two groups. |

| \| **PNP/PNP** \| 30 \| 32 \| 34 \| 36 \| 38 \| 40 \| \| --- \| --- \| --- \| --- \| --- \| --- \| --- \| \| 28 \| **0.96** \| **1.52** \| **1.84** \| **2.27** \| **2.64** \| **2.99** \| \| 30 \|  \| **0.88** \| **1.19** \| **1.62** \| **1.99** \| **2.34** \| \| 32 \|  \|  \| **0.63** \| **1.05** \| **1.43** \| **1.78** \| \| 34 \|  \|  \|  \| **0.74** \| **1.11** \| **1.46** \| \| 36 \|  \|  \|  \|  \| **0.69** \| **1.03** \| \| 38 \|  \|  \|  \|  \|  \| **0.64** \| |
| --- | --- | --- | --- | --- | --- | --- | --- | --- | --- | --- | --- | --- | --- | --- | --- | --- | --- | --- | --- | --- | --- | --- | --- | --- | --- | --- | --- | --- | --- | --- | --- | --- | --- | --- | --- | --- | --- | --- | --- | --- | --- | --- | --- | --- | --- | --- | --- | --- | --- |
| **Table S4.** Differences in the ablation area in millimeters squared between pressure groups for clots composed of more than 75% red blood cells (N = 7 per peak negative pressure). The peak negative pressure (PNP) is reported in units of MPa. Bold values indicate significant differences between the two groups. |

| \| **PNP/PNP** \| 30 \| 32 \| 34 \| 36 \| 38 \| 40 \| \| --- \| --- \| --- \| --- \| --- \| --- \| --- \| \| 28 \| 0.22 \| 0.27 \| 0.32 \| **0.37** \| **0.42** \| **0.47** \| \| 30 \|  \| 0.22 \| 0.27 \| 0.32 \| **0.37** \| **0.41** \| \| 32 \|  \|  \| 0.22 \| 0.27 \| 0.31 \| **0.36** \| \| 34 \|  \|  \|  \| 0.21 \| 0.26 \| 0.31 \| \| 36 \|  \|  \|  \|  \| 0.21 \| 0.26 \| \| 38 \|  \|  \|  \|  \|  \| 0.21 \| |
| --- | --- | --- | --- | --- | --- | --- | --- | --- | --- | --- | --- | --- | --- | --- | --- | --- | --- | --- | --- | --- | --- | --- | --- | --- | --- | --- | --- | --- | --- | --- | --- | --- | --- | --- | --- | --- | --- | --- | --- | --- | --- | --- | --- | --- | --- | --- | --- | --- | --- |
| **Table S5.** Differences in the ablation area in millimeters squared between pressure groups for clots composed of more than 75% fibrin (N = 7 per peak negative pressure). The peak negative pressure (PNP) is reported in units of MPa. Bold values indicate significant differences between the two groups. |

| \| **PNP/PNP** \| 30 \| 32 \| 34 \| 36 \| 38 \| 40 \| \| --- \| --- \| --- \| --- \| --- \| --- \| --- \| \| 28 \| **0.65** \| **0.95** \| **1.23** \| **1.49** \| **1.72** \| **1.95** \| \| 30 \|  \| 0.6 \| **0.89** \| **1.15** \| **1.38** \| **1.60** \| \| 32 \|  \|  \| 0.59 \| **0.85** \| **1.08** \| **1.30** \| \| 34 \|  \|  \|  \| 0.56 \| **0.79** \| **1.02** \| \| 36 \|  \|  \|  \|  \| 0.54 \| **0.76** \| \| 38 \|  \|  \|  \|  \|  \| 0.53 \| |
| --- | --- | --- | --- | --- | --- | --- | --- | --- | --- | --- | --- | --- | --- | --- | --- | --- | --- | --- | --- | --- | --- | --- | --- | --- | --- | --- | --- | --- | --- | --- | --- | --- | --- | --- | --- | --- | --- | --- | --- | --- | --- | --- | --- | --- | --- | --- | --- | --- | --- |
| **Table S6.** Differences in the ablation area in millimeters squared between pressure groups for clots composed of between 25 and 75% red blood cells (N = 8 per peak negative pressure). The pressure is reported in units of MPa. Bold values indicate significant differences between the two groups. |

Comparison of Fibrin Degradation Production Between Histotripsy Pulse Pressures

The total generation of fibrin degradation products was tabulated for each set of investigated conditions (clot environment and histotripsy exposure). Here, the clot environment was based on either baseline conditions (i.e. no histotripsy exposure) or based on changes to the clot structure due to histotripsy exposure (i.e. Monte Carlo predictions based on the application of 1,000 histotripsy pulses with peak negative pressures of 28 to 40 MPa). To determine differences in the concentration of fibrin degradation products between histotripsy pulse peak negative pressures, ANOVA was conducted, using Tukey’s HSD to determine differences between groups (*p* < 0.05). Analysis was conducted for all clots, and each clot subgroup.

| \| **PNP/PNP** \| 28 \| 30 \| 32 \| 34 \| 36 \| 38 \| 40 \| \| --- \| --- \| --- \| --- \| --- \| --- \| --- \| --- \| \| 0 \| 2.33 \| 1.86 \| 1.93 \| **3.65** \| **3.81** \| **3.95** \| **4.09** \| \| 28 \|  \| 1.23 \| 1.30 \| **3.02** \| **3.19** \| **3.32** \| **3.46** \| \| 30 \|  \|  \| 1.78 \| **3.50** \| **3.67** \| **3.80** \| **3.94** \| \| 32 \|  \|  \|  \| **3.43** \| **3.59** \| **3.73** \| **3.87** \| \| 34 \|  \|  \|  \|  \| 1.87 \| 2.01 \| 2.15 \| \| 36 \|  \|  \|  \|  \|  \| 1.85 \| 1.98 \| \| 38 \|  \|  \|  \|  \|  \|  \| 1.85 \| |
| --- | --- | --- | --- | --- | --- | --- | --- | --- | --- | --- | --- | --- | --- | --- | --- | --- | --- | --- | --- | --- | --- | --- | --- | --- | --- | --- | --- | --- | --- | --- | --- | --- | --- | --- | --- | --- | --- | --- | --- | --- | --- | --- | --- | --- | --- | --- | --- | --- | --- | --- | --- | --- | --- | --- | --- | --- | --- | --- | --- | --- | --- | --- | --- | --- |
| **Table S7.** Differences in the degree of fibrinolysis relative to the baseline value between pressure groups for all clot groups. The pressure is reported in units of MPa. Bold values indicate significant differences between the two groups. |

| \| **PNP/PNP** \| 28 \| 30 \| 32 \| 34 \| 36 \| 38 \| 40 \| \| --- \| --- \| --- \| --- \| --- \| --- \| --- \| --- \| \| 0 \| 5.14 \| 5.25 \| 5.36 \| **10.66** \| **11.08** \| **11.42** \| **11.72** \| \| 28 \|  \| 5.14 \| 5.25 \| **10.55** \| **10.97** \| **11.31** \| **11.62** \| \| 30 \|  \|  \| 5.13 \| **10.43** \| **10.85** \| **11.19** \| **11.50** \| \| 32 \|  \|  \|  \| **10.33** \| **10.75** \| **11.08** \| **11.39** \| \| 34 \|  \|  \|  \|  \| 5.44 \| 5.78 \| 6.09 \| \| 36 \|  \|  \|  \|  \|  \| 5.36 \| 5.67 \| \| 38 \|  \|  \|  \|  \|  \|  \| 5.33 \| |
| --- | --- | --- | --- | --- | --- | --- | --- | --- | --- | --- | --- | --- | --- | --- | --- | --- | --- | --- | --- | --- | --- | --- | --- | --- | --- | --- | --- | --- | --- | --- | --- | --- | --- | --- | --- | --- | --- | --- | --- | --- | --- | --- | --- | --- | --- | --- | --- | --- | --- | --- | --- | --- | --- | --- | --- | --- | --- | --- | --- | --- | --- | --- | --- | --- |
| **Table S8.** Differences in the degree of fibrinolysis relative to the baseline value between pressure groups for clots with more than 75% red blood cells. The pressure is reported in units of MPa. Bold values indicate significant differences between the two groups. |

| \| **PNP/PNP** \| 28 \| 30 \| 32 \| 34 \| 36 \| 38 \| 40 \| \| --- \| --- \| --- \| --- \| --- \| --- \| --- \| --- \| \| 0 \| 0.13 \| **0.18** \| **0.21** \| **0.24** \| **0.28** \| **0.32** \| **0.40** \| \| 28 \|  \| 0.14 \| **0.17** \| **0.20** \| **0.34** \| **0.28** \| **0.36** \| \| 30 \|  \|  \| 0.12 \| 0.15 \| **0.18** \| **0.22** \| **0.30** \| \| 32 \|  \|  \|  \| 0.11 \| 0.15 \| **0.19** \| **0.27** \| \| 34 \|  \|  \|  \|  \| 0.12 \| 0.15 \| **0.24** \| \| 36 \|  \|  \|  \|  \|  \| 0.12 \| **0.20** \| \| 38 \|  \|  \|  \|  \|  \|  \| 0.16 \| |
| --- | --- | --- | --- | --- | --- | --- | --- | --- | --- | --- | --- | --- | --- | --- | --- | --- | --- | --- | --- | --- | --- | --- | --- | --- | --- | --- | --- | --- | --- | --- | --- | --- | --- | --- | --- | --- | --- | --- | --- | --- | --- | --- | --- | --- | --- | --- | --- | --- | --- | --- | --- | --- | --- | --- | --- | --- | --- | --- | --- | --- | --- | --- | --- | --- |
| **Table S9.** Differences in the degree of fibrinolysis relative to the baseline value between pressure groups for clots composed of more than 75% fibrin. The pressure is reported in units of MPa. Bold values indicate significant differences between the two groups. |

| \| **PNP/PNP** \| 28 \| 30 \| 32 \| 34 \| 36 \| 38 \| 40 \| \| --- \| --- \| --- \| --- \| --- \| --- \| --- \| --- \| \| 0 \| **0.11** \| **0.17** \| **0.24** \| **0.31** \| **0.36** \| **0.41** \| **0.45** \| \| 28 \|  \| **0.11** \| **0.18** \| **0.25** \| **0.30** \| **0.35** \| **0.39** \| \| 30 \|  \|  \| **0.12** \| **0.19** \| **0.24** \| **0.29** \| **0.33** \| \| 32 \|  \|  \|  \| **0.12** \| **0.17** \| **0.21** \| **0.26** \| \| 34 \|  \|  \|  \|  \| **0.10** \| **0.14** \| **0.19** \| \| 36 \|  \|  \|  \|  \|  \| **0.09** \| **0.14** \| \| 38 \|  \|  \|  \|  \|  \|  \| **0.09** \| |
| --- | --- | --- | --- | --- | --- | --- | --- | --- | --- | --- | --- | --- | --- | --- | --- | --- | --- | --- | --- | --- | --- | --- | --- | --- | --- | --- | --- | --- | --- | --- | --- | --- | --- | --- | --- | --- | --- | --- | --- | --- | --- | --- | --- | --- | --- | --- | --- | --- | --- | --- | --- | --- | --- | --- | --- | --- | --- | --- | --- | --- | --- | --- | --- | --- |
| **Table S10.** Differences in the degree of fibrinolysis relative to the baseline value between pressure groups for clots composed of between 25 and 75% red blood cells. The pressure is reported in units of MPa. Bold values indicate significant differences between the two groups. |

Influence of histotripsy pulse pressure on FDTD calculation of rt-PA distributions:

Representative examples of rt-PA distribution within the clot and the corresponding degree of fibrinolysis are shown in **Fig. S5** relative to the histotripsy pulse peak negative pressure. In the absence of histotripsy, the rt-PA concentration was symmetrically distributed. Beyond a distance of ~ 2 mm from the catheter, PAI-1 quenched the thrombolytic drug and minimal fibrin degradation products were observed. Histotripsy results in rt-PA uniformly distributed within the focal region due to the increased diffusivity. The resulting effect approximated a larger catheter, with an increased high concentration of rt-PA throughout the clot. Consequently, the area and degree of fibrin degradation production increased as the peak negative pressure of the histotripsy pulse increased.

|  |
| --- |
| **Fig. S5**. Monte Carlo predictions of clot structure after the application of 1,000 histotripsy pulses (top row), and the corresponding distribution of rt-PA within the ablated clot assessed via the perfusion-diffusion equation (middle row row). The computed distribution of fibrin degradation products (FDP) resulting from a 20 min rt-PA exposure is shown in the bottom row. Blue pixels correspond to the area of the catheter. The peak negative pressure of the histotripsy pulse is noted at the top of each column. The black line in the top row corresponds to a 1 mm distance. |

Number of pulses required to maximize histotripsy ablation:

Enhanced fibrinolysis was due to an increased distribution of thrombolytic drug within ablated areas (**Fig. 8**). The ablation area was found to increase rapidly during the initial stages of histotripsy exposure, as indicated in **Fig. 6**. Beyond the application of a critical number of pulses, there was a reduction in the growth of the ablation zone. To determine this critical exposure duration, a piecewise linear fit was applied to the ablation area as a function of the number of applied pulses using a least-squares criterion (**Fig. S6**). The number of pulses required to maximize the ablation area was defined as the inflection point in the piecewise linear function (9), and is reported in **Table S11** for each thrombus subgroup at the histotripsy pulse peak negative pressure necessary to enhance fibrinolysis: 28 MPa for red blood cell-dominant, 30 MPa for Half-Half, and 36 MPa for fibrin-dominant (**Fig. 9**).

|  |
| --- |
| **Fig. S6**. Representative ablation area relative to the number of applied histotripsy pulses (solid line) and piecewise linear fit (dashed line). Error bars represent the standard deviation of the ablation area for ten iterations of the Monte Carlo calculation. The inflection point in the piecewise linear fit represents the number of pulses required to maximize the ablation area. |

| \| **Thrombus Subgroup** \| **Number of Pulses to Maximize Ablation** \| \| --- \| --- \| \| Red Blood Cell Dominant \| 160 ± 52 \| \| Half-Half \| 200 ± 63 \| \| Fibrin Dominant \| 390 ± 76 \| |
| --- | --- | --- | --- | --- | --- | --- | --- | --- |
| **Table S11.** Number of applied histotripsy pulses required to maximize the ablation at each peak negative pressure necessary for enhanced fibrinolysis:28 MPa for red blood cell dominant (N = 7), 30 MPa for Half-Half (N = 8), and 36 MPa for fibrin dominant (N = 7). |

**References**

1. Kinnunen M, Kauppila A, Karmenyan A, Myllylä R. Effect of the size and shape of a red blood cell on elastic light scattering properties at the single-cell level. Biomed Opt Express. 2011 Jul;2(7):1803–14.

2. Bader KB, Haworth KJ, Shekhar H, Maxwell AD, Peng T, McPherson DD, et al. Efficacy of histotripsy combined with rt-PA in vitro. Phys Med Biol. 2016;61(14):5253–74.

3. Maxwell AD, Haworth KJ, Holland CK, Hendley SA, Kreider W, Bader KB. Design and Characterization of an Ultrasound Transducer for Combined Histotripsy-Thrombolytic Therapy. IEEE Trans Ultrason Ferroelectr Freq Control [Internet]. 2022 Jan;69(1):156–65. Available from: https://ieeexplore.ieee.org/document/9540861/

4. Hendley SA, Dimov A, Bhargava A, Snoddy E, Mansour D, Afifi RO, et al. Assessment of histological characteristics, imaging markers, and rt-PA susceptibility of ex vivo venous thrombi. Sci Rep [Internet]. 2021 Dec 23;11(1):22805. Available from: https://www.nature.com/articles/s41598-021-02030-7

5. Anthony GJ, Bollen V, Hendley S, Antic T, Sammet S, Bader KB. Assessment of histotripsy-induced liquefaction with diagnostic ultrasound and magnetic resonance imaging in vitro and ex vivo. Phys Med Biol. 2019 May;64(9):095023--.

6. Maxwell AD, Wang TY, Yuan L, Duryea AP, Xu Z. A tissue phantom for visualization and measurement of ultrasound-induced cavitation damage. Ultrasound Med Biol. 2010;36(12):2132–43.

7. Bollen V, Hendley SA, Paul JD, Maxwell AD, Haworth KJ, Holland CK, et al. In Vitro Thrombolytic Efficacy of Single-and Five-Cycle Histotripsy Pulses and rt-PA. Ultrasound Med Biol. 2020 Feb;46(2):336–49.

8. Bear J. Dynamics of fluids in porous media. First. New York, NY: American Elsevier Publishing Compand; 1972. 621 p.

9. Bhargava A, Huang S, McPherson DD, Bader KB. Assessment of bubble activity generated by histotripsy combined with echogenic liposomes. Phys Med Biol [Internet]. 2022 Nov 7;67(21):215015. Available from: https://iopscience.iop.org/article/10.1088/1361-6560/ac994f
